# Polyphosphate Copper Nanospheres Drive Mitochondrial Metabolic Reprogramming via Calcium Phase Separation for Self‐Sensitizing Cuproptosis

**DOI:** 10.1002/advs.78368

**Published:** 2026-10-22

**Authors:** Jing Wang, Xinyang Zhao, Zifeng Huang, Jingyi Zhang, Yucheng Tao, Yong Huang, Zijian Guo, Lei Dong, Xiuxiu Wang, Jing Zhao, Wei Wei

**Affiliations:** ^1^ State Key Laboratory of Coordination Chemistry School of Chemistry Nanjing University Nanjing People's Republic of China; ^2^ Chemistry and Biomedicine Innovation Center (ChemBIC) School of Chemistry Nanjing University Nanjing People's Republic of China; ^3^ School of Life Sciences Nanjing University Nanjing People's Republic of China; ^4^ Department of Chemical and Biomolecular Engineering National University of Singapore Singapore Singapore; ^5^ Department of Chemistry The Hong Kong University of Science and Technology Kowloon Hong Kong SAR People's Republic of China; ^6^ Nanchuang (Jiangsu) Institute of Chemistry and Health Sino‐Danish Ecolife Science Industrial Incubator Nanjing People's Republic of China; ^7^ Wuxi Xishan NJU Institute of Applied Biotechnology Wuxi People's Republic of China

**Keywords:** cuproptosis, liquid–liquid phase separation, mitochondrial metabolism, polyphosphate

## Abstract

Precise spatiotemporal control of intracellular Ca^2+^ signaling remains a major bottleneck in reversing tumor glycolytic dominance and reactivating mitochondrial therapies. To overcome this limitation, we developed a polyphosphate (polyP)‐engineered nanoplatform that exploits liquid‐liquid phase separation (LLPS) to drive calcium‐mediated metabolic reprogramming. We discovered that FITC‐polyP coacervates with Ca^2+^ to form biomolecular condensates that function as localized Ca^2+^ signaling microdomains. By selectively activating key mitochondrial enzymes, these microdomains shift tumor cells toward a mitochondria‐dependent metabolic phenotype. Crucially, disturbing condensate formation via Ca^2+^ chelation or enzymatic degradation prevents this transition, confirming polyP/Ca^2+^ condensates as the indispensable master regulator of mitochondrial metabolic activation. Sustained mitochondrial reactivation creates a primed cellular state that sensitizes tumor cells to cuproptosis. This work establishes a resourceful self‐sensitizing nanoplatform that utilizes polyP‐induced phase separation to precisely reprogram tumor metabolism and activate cuproptosis, highlighting polyP‐induced LLPS as a novel strategy for targeted cancer therapy.

## Graphical abstract

This study developed polyphosphate (polyP)‐based nanostructures that enable calcium‐mediated metabolic reprogramming via liquid–liquid phase separation (LLPS). Released polyP undergoes LLPS with Ca^2+^, suppressing glycolytic flux and enhancing mitochondrial metabolism effectively. This creates an ideal “metabolically sensitized” microenvironment for cuproptosis, triggering a synergistic effect between mitochondrial metabolic activation and cuproptosis.

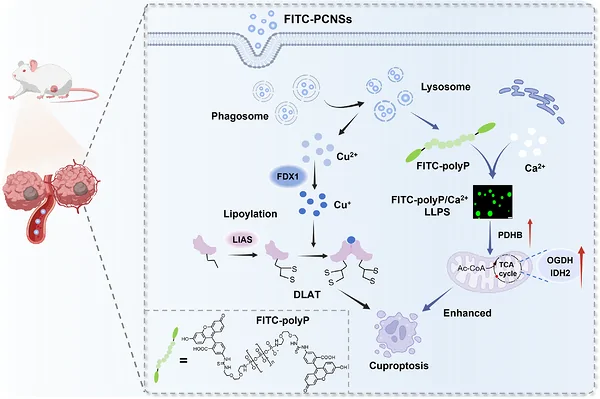

## 1 Introduction

Certain cancer cells exhibit remarkable metabolic reprogramming that distinguishes them from normal cells, presenting novel opportunities to develop therapeutic strategies that target tumor‐specific metabolic vulnerabilities [1, 2, 3]. Among these metabolic alterations, the preferential utilization of aerobic glycolysis over mitochondrial oxidative phosphorylation (OXPHOS) even under normoxic conditions represents a critical survival strategy for many malignancies [4, 5, 6]. This glycolytic phenotype confers dual evolutionary advantages: (1) Sustained adenosine triphosphate (ATP) generation under fluctuating oxygen tension through oxygen‐independent pathways, enabling adaptation to hypoxic tumor microenvironments; (2) Enhanced flux of glycolytic intermediates toward anabolic pathways, supplying essential building blocks for nucleic acid, lipid, and protein biosynthesis to support rapid proliferation and metastatic spread [7, 8, 9, 10]. This metabolic alteration creates therapeutic dilemmas by simultaneously desensitizing tumors to mitochondria‐targeted therapies and fostering lactate‐mediated immunosuppression through tumor microenvironment remodeling [11, 12, 13, 14].

Within this context, achieving precise modulation of mitochondrial metabolic function becomes paramount. Ca^2+^, as a ubiquitous second messenger, not only regulates diverse biological processes, including cell proliferation, death, and immune responses, but also serves as a critical regulator of mitochondrial metabolism [15]. The accumulation of Ca^2+^ within mitochondria activates Ca^2+^‐sensitive dehydrogenases in the tricarboxylic acid (TCA) cycle, a process essential for optimal mitochondrial function [16, 17]. Consequently, developing technological systems capable of precisely modulating mitochondrial Ca^2+^ homeostasis represents a key strategy for effectively intervening in the mitochondrial metabolism of tumor cells. However, achieving spatiotemporally precise control over Ca^2+^ signaling and its downstream metabolic effects within the complex intracellular milieu remains a significant challenge. Critically, emerging evidence suggests that liquid‐liquid phase separation (LLPS), a process driving the formation of biomolecular condensates, plays a fundamental role in organizing and compartmentalizing key signaling components, including those regulating Ca^2+^ dynamics and metabolic enzymes [18, 19, 20]. This raises the compelling possibility that specific LLPS events could act as regulatory hubs, locally concentrating Ca^2+^ and its effectors to modulate core mitochondrial metabolic pathways with high fidelity, thereby offering a novel mechanism for metabolic reprogramming.

The central role of LLPS in key cellular processes, including transcriptional regulation and signal transduction, has been well established. Pioneering work by Alberti, Hyman, and colleagues has been instrumental in elucidating how eukaryotic cells exploit LLPS [21, 22]. Given that electrostatic interactions represent a primary driving force for LLPS, we focus on polyphosphate (polyP), an evolutionarily conserved, linear inorganic polymer composed of phosphate units linked by high‐energy phosphoanhydride bonds [23, 24, 25]. As one of the most densely negatively charged polyanions within the cell (second only to nucleic acids and ATP), polyP, with its potent electrostatic potential, emerges as an ideal candidate for regulating the phase behavior of biomolecules [26, 27, 28]. Research has confirmed that polyP possesses significant phase separation capabilities and plays critical roles in diverse biological processes [29]. Jakob and coworkers discovered that in bacteria, polyP and the RNA chaperone Hfq cooperatively drive the formation of heterochromatin‐like domains via phase separation, thereby regulating DNA damage [30]. Racki, Deniz, and coworkers demonstrated that phase separation of polyP with Mg^2+^ can remodel the spatial conformation of DNA [31]. Furthermore, Shiraki and coworkers revealed that the dynamic regulation of monovalent ion concentration within living cells is a key mechanism governing polyP aggregate formation and morphological changes [32]. Based on the high negative charge density of polyP and its well‐established role in LLPS, we propose that polyP may exert spatiotemporally precise regulation over Ca^2+^ signaling and metabolic pathways at the subcellular level through LLPS, and offer a novel therapeutic avenue for developing LLPS‐based strategies to intervene in tumor metabolism.

In this study, we engineered a fluorescein isothiocyanate (FITC)‐ labeled FITC‐polyP‐based nanoplatform that harnesses LLPS for spatiotemporal metabolic control. Specifically, we discovered that FITC‐polyP with high‐energy phosphoanhydride bonds undergoes LLPS with Ca^2+^ in the cell, which subsequently triggers a metabolic shift, suppressing glycolytic flux while amplifying OXPHOS in tumor cells. And, interestingly, this shift further drives tumor cells toward self‐sensitizing cuproptosis (Scheme 1). When disturbing the formation of the FITC‐polyP/Ca^2+^ condensate through Ca^2+^ chelation or enzymatic inhibition, this energy metabolic shift can be significantly prevented, confirming that the LLPS condensate formed by FITC‐polyP/Ca^2+^ is the key regulator of the mitochondrial metabolism transition. Concurrently, this metabolic remodeling reduces lactate secretion, which is accompanied by enhanced immune cell infiltration in the tumor microenvironment. This work elucidates the “Ca^2+^ phase separation to mitochondrial metabolic activation to cuproptosis sensitization” cascade mechanism, providing not only an innovative strategy for tumor metabolic intervention but also deepening the theoretical understanding of phase separation in cell fate regulation.

**SCHEME 1:**
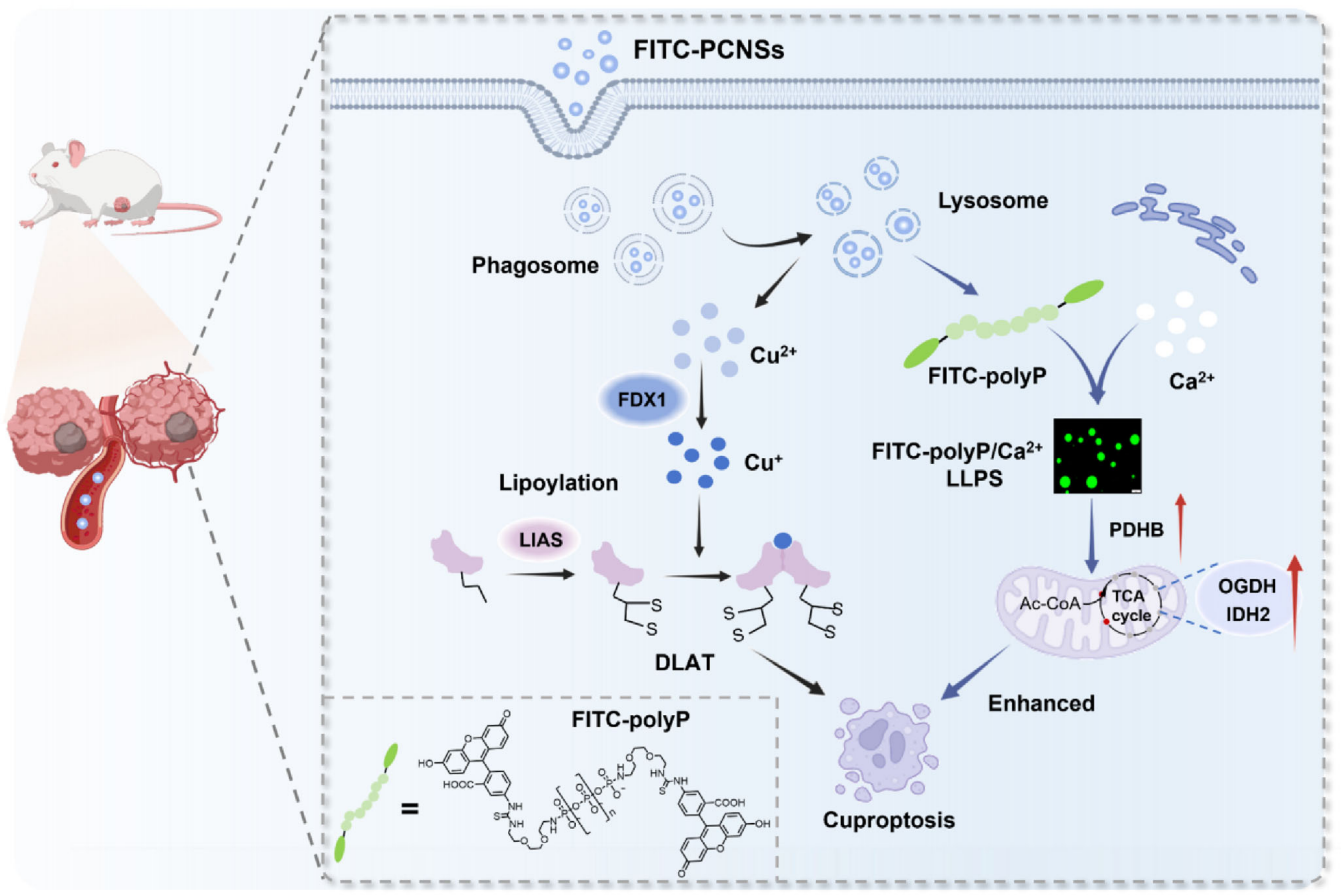
Schematic illustration of FITC‐PCNSs reprogramming tumor cell mitochondrial metabolism by phase separation to achieve cuproptosis sensitization.

## 2 Results and Discussion

### 2.1 Rational Design and Controlled Synthesis of polyP‐Functionalized Nanoplatforms

In mammals, polyP functions in blood coagulation, modulation of inflammatory responses, and participation in other signaling pathways through its storage and release by blood platelets in circulation [33, 34]. However, due to its high‐density surface negative charges, polyP exhibits inherently poor cellular permeability, limiting direct intracellular functionality. To investigate its intracellular roles, we designed polyP‐functionalized nanoplatforms through interactions between polyP and metal ions under tunable reaction conditions. These nanostructures display enhanced biocompatibility and cellular penetrability while enabling controlled release of polyP and metal ions as bioactive payloads. Following the referenced methodologies [35, 36], we first synthesized fluorescently tagged FITC‐polyP through site‐specific covalent conjugation between FITC and terminal phosphate moieties of polyP (Figures S1–S7). Subsequently, fluorescent polyP‐based nanostructures were fabricated via electrostatic interactions between phosphate groups and metal ions (Cu^2+^, Zn^2+^, Mn^2+^, Ga^3+^, Mg^2+^, Ca^2+^), as shown in Figure 1a,b; Figure S8.

**FIGURE 1:**
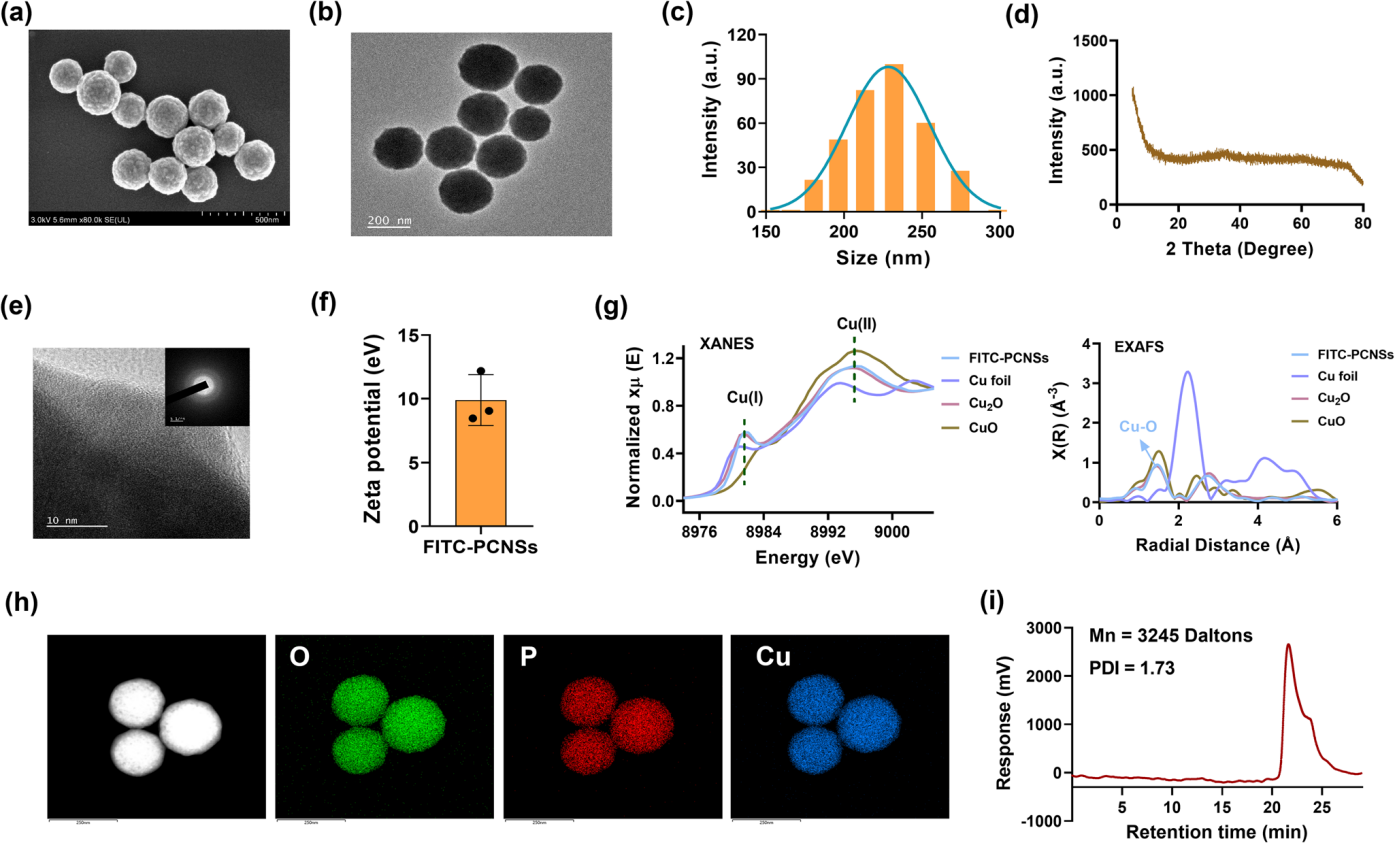
Morphology and structural characterization of the FITC‐PCNSs. (a) A typical SEM image of the FITC‐PCNSs. Scale bar: 500 nm. (b) A typical TEM image of the FITC‐PCNSs. Scale bar: 200 nm. (c) DLS analysis of the FITC‐PCNSs. (d) XRD results of the FITC‐PCNSs. (e) HRTEM image of the FITC‐PCNSs with the inset showing the SAED pattern. Scale bar: 10 nm. (f) Zeta potential of FITC‐PCNSs. (g) Cu K‐edge XANES spectra (left) and EXAFS spectra (right). (h) STEM images and elemental mapping of FITC‐PCNSs. Scale bar: 250 nm. (i) GPC curve of FITC‐polyP released from FITC‐PCNSs after 24 h. All data are displayed as mean ± standard deviation (*n* = 3).

All nanostructures exhibited an amorphous nanoparticulate morphology (Figure S8). Notably, transmission electron microscopy (TEM) and scanning electron microscopy (SEM) characterization of FITC‐polyP‐Cu (FITC‐PCNSs) revealed a spherical structure with a uniform lateral size distribution of 150–300 nm, as confirmed by dynamic light scattering (DLS) (Figure 1a–c). The amorphous nature of the material was established through PXRD analysis and high‐resolution TEM (HRTEM) images, which collectively demonstrated a disordered atomic arrangement (Figure 1d,e). Surface charge analysis via zeta potential measurements indicated a mild positive charge, suggesting that it can be well taken up by cells (Figure 1f). Elemental mapping using Energy‐dispersive X‐ray spectroscopy (EDXS) confirmed the homogeneous distribution of P, Cu, and O throughout the nanospheres (Figure 1h). X‐ray photoelectron spectroscopy (XPS) analysis of the O*1s* revealed a near‐equimolar ratio of Cu─O to P─O bonds (Figure S9). The Cu K‐edge XANES spectra of FITC‐PCNSs showed that the Cu (I) and Cu (II) peaks were highlighted at ∼ 8981 eV and 8995 eV, respectively. Likewise, the extended X‐ray absorption fine structure (EXAFS) of FITC‐PCNSs exhibited Cu─O interatomic distances of 1.47 Å. The coordination number for Cu─O in the first shell was 1.8 (Figure 1g). FITC‐PCNSs and unlabeled PCNSs exhibited nearly identical morphology, size distribution, surface potential, and copper loading efficiencies between the two nanospheres (Figure S10), proving that FITC functionalization does not alter the intrinsic physicochemical characteristics of PCNSs.The nanospheres exhibited fluorescence emission maxima at 525 nm, confirming retention of FITC photophysical properties (Figure S11a). The nanostructures demonstrated structural integrity at physiological pH 7.4, while exhibiting progressive liberation of Cu^2+^ and FITC‐polyP components under lysosomal‐mimetic acidic conditions (pH 5.5) (Figure S11b). Gel permeation chromatography (GPC) quantification confirmed that FITC‐polyP maintained its molecular integrity after release, achieving a sustained release equilibrium within 24 h under these acidic conditions (Figures 1i; Figures S12–S19).

### 2.2 FITC‐PCNSs Promote a Metabolic Shift from Glycolysis to Mitochondrial Metabolism in Tumor Cells

To evaluate the metabolic regulatory role of polyP‐based nanomaterials, we systematically assessed their impact on glycolytic capacity and ATP production in 4T1 tumor cells. Initial screening revealed that all structures consistently suppressed lactate secretion and boosted OXPHOS‐derived ATP production (Figure 2a–c; Figure S20). Notably, FITC‐PCNSs exhibited superior efficacy in both lactate inhibition and tumor cell killing (Figures S20 and S21 and Table S1). Given that heightened mitochondrial metabolism sensitizes cells to cuproptosis, FITC‐PCNSs were selected as the model system to decipher the specific mechanisms of metabolic reprogramming.

**FIGURE 2:**
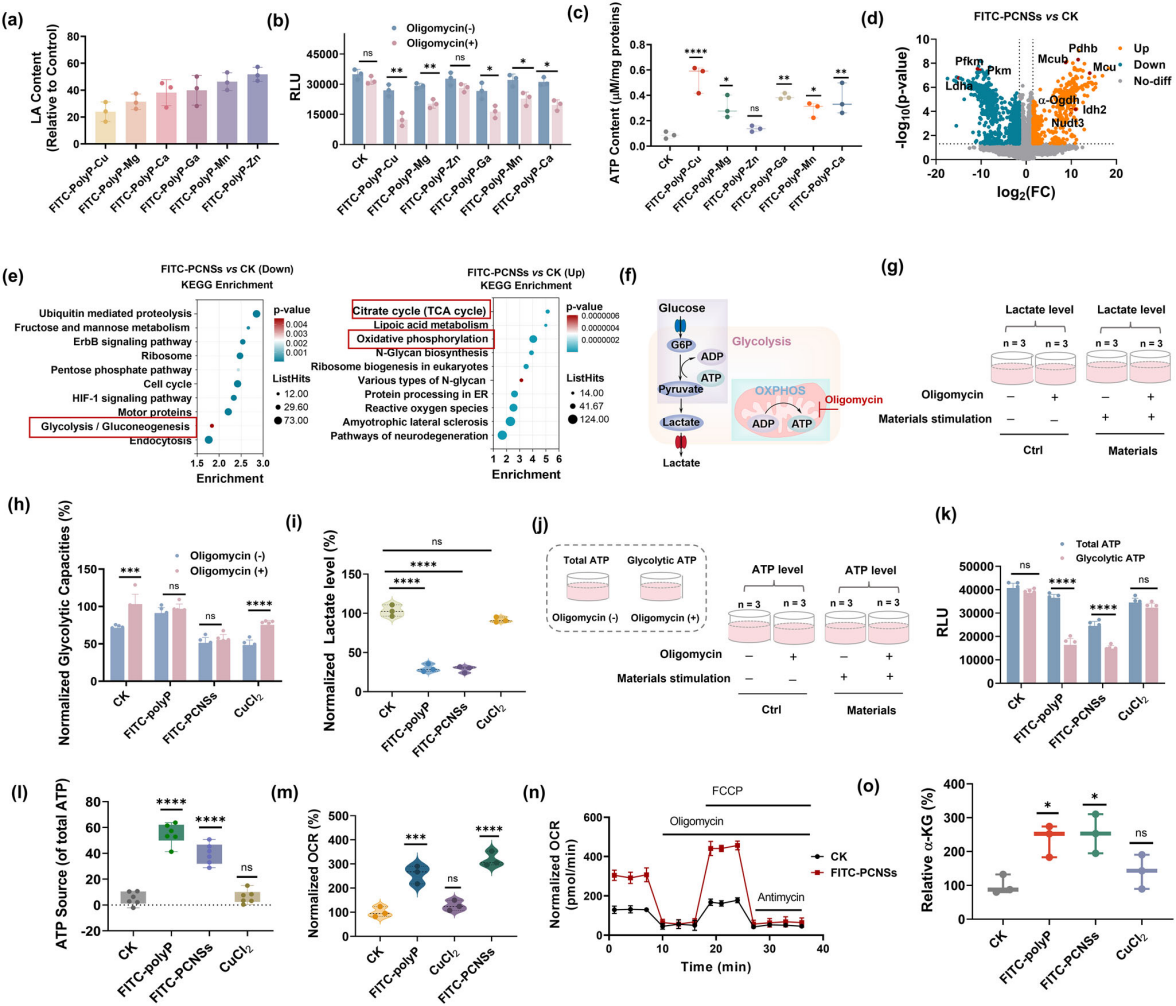
FITC‐PCNSs promote the reprogramming of cellular metabolism from glycolysis to OXPHOS. (a) Inhibition of lactate production in 4T1 tumor cells mediated by various polyP‐based nanostructures. (b) Total ATP production levels in cells treated with various polyP‐based materials in the presence or absence of oligomycin, and (c) corresponding mitochondrial ATP generation levels. (d) Volcano plot of differentially expressed proteins in 4T1 cells treated with FITC‐PCNSs compared with the blank control. (e) KEGG pathway analysis of downregulated (left) and upregulated (right) proteins. (f) Schematic illustration of ATP synthesis inhibition. The pathway for ATP synthesis via OXPHOS was inhibited following treatment with oligomycin. During this time, lactate production was measured as an indicator to assess the degree of glycolytic hyperactivity in the alternative ATP‐generating pathway. (g) Schematic diagram of the experimental setup for the assessment of cellular glycolytic capacity. (h) Changes in cellular glycolytic capacity after treatment with FITC‐polyP, CuCl_2_, and FITC‐PCNSs. (i) Normalized lactate levels after treatment with FITC‐polyP, CuCl_2_, and FITC‐PCNSs. (j) Schematic diagram of the experimental setup for the evaluation of ATP production by different metabolic pathways. The levels of total ATP and ATP production via the glycolytic pathway (k) and ATP production via OXPHOS (l) after treatment with FITC‐polyP, CuCl_2_, and FITC‐PCNSs. (m) Normalized OCR after treatment with FITC‐polyP, CuCl_2_, and FITC‐PCNSs. (n) Mitochondrial respiratory capacity measured via normalized OCR. (o) The level of α‐KG after treatment with FITC‐polyP, CuCl_2_, and FITC‐PCNSs. The concentrations of all materials in figures h, i, k, l, and m are: FITC‐polyP (0.057 µm, calculated), CuCl_2_ (100 µm), and FITC‐PCNSs (25 µg mL^−1^). All data are displayed as mean ± standard deviation (*n* = 3). ^*^p < 0.05, ^**^p < 0.01, ^***^p < 0.001, and ^****^ *p* < 0.0001; “ns” indicates no significant difference; data in Figure 2b,h,k were analyzed by Student's *t*‐test; data in Figure 2c,i,l,m were analyzed by One‐Way ANOVA.

Before evaluating metabolic regulation, we verified the cellular uptake mechanism and intracellular fate of FITC‐PCNSs. Uptake kinetics showed a continuous increase in intracellular copper levels over 24 h (reaching 16.49 µg/10^7^ cells; Figure S22). Pretreatment with Pitstop 2 significantly attenuated intracellular FITC fluorescence, confirming clathrin‐mediated endocytosis as the primary entry pathway (Figure S23). Corresponding confocal laser scanning microscopy (CLSM) analysis demonstrated selective co‐localization with lysosomes, but not with mitochondria, the endoplasmic reticulum (ER), or the Golgi apparatus (Figure S24). Driven by the acidic lysosomal lumen (∼pH 5.5) and consistent with the in vitro release profile (Figure S11b), internalized FITC‐PCNSs readily dissociate within lysosomes to release Cu^2^^+^ and FITC‐polyP.

Next, we performed proteomic analysis to elucidate the metabolic impact of FITC‐PCNSs. Proteomic profiling revealed a global suppression of the glycolytic pathway alongside significant enrichment of the TCA cycle and OXPHOS pathways (Figure 2e). At the protein level, key glycolytic enzymes, such as phosphofructokinase (PFK), pyruvate kinase M2 (PKM2), and lactate dehydrogenase A (LDHA), were markedly downregulated. In contrast, critical TCA cycle dehydrogenases, including α‐ketoglutarate dehydrogenase (α‐OGDH) and isocitrate dehydrogenase 2 (IDH2), were significantly upregulated (Figure 2d). Functional assays further confirmed this molecular level shift from glycolysis to mitochondrial respiration. Specifically, FITC‐PCNSs treatment led to a 7.84‐fold reduction in extracellular lactate and a 7.89‐fold decline in glycolytic ATP, which was contrasted by a 9.74‐fold surge in mitochondrial ATP production (Figure 2f–l). Meanwhile, CuCl_2_ treatment induced no appreciable change. This respiratory activation was further corroborated by a 3.18‐fold increase in oxygen consumption rate (OCR), accompanied by elevated NADH/(NADH + NAD^+^) ratios and α‐KG levels (Figure 2m–o; Figure S25). Notably, lipofection‐mediated intracellular delivery of free FITC‐polyP (0.057 µm at 24 h; Figures S26 and S36) independently triggered comparable metabolic reprogramming, reducing glycolytic capacity by 5.86‐fold while boosting mitochondrial ATP generation by 4.89‐fold (Figure 2h,i,k,l; Figures S27 and S28). Together, these findings validate the potent metabolic regulatory function of FITC‐PCNSs and establish FITC‐polyP as the indispensable active component driving this metabolic shift.

### 2.3 FITC‐polyP/Ca^2+^ Condensates Serve as the Critical Determinant in Metabolic Reprogramming

Proteomic analysis revealed upregulated expression of the mitochondrial calcium uniporter (MCU), suggesting potential modulation of intracellular Ca^2+^ homeostasis (Figure 2d). Concurrently, expression levels of inositol trisphosphate (IP3) and its receptor (IP3R) were markedly elevated. IP3 activates ER‐localized IP3R channels, thereby facilitating ER Ca^2+^ release to regulate nucleocytoplasmic and organellar Ca^2+^ dynamics [37, 38, 39]. Following FITC‐PCNSs treatment, intracellular IP3 levels increased 7.70‐fold relative to controls, accompanied by a 4.51‐fold upregulation of IP3R and a concomitant increase in cytosolic Ca^2+^ fluorescence (Figure 3a,b). Interestingly, fluorescence imaging further captured punctate Ca^2+^ signals that strongly co‐localized with FITC‐polyP (PCC = 0.87, Figure 3d; Figures S29 and S30). Subcellular tracking indicated that these condensates were localized within the cytosol rather than residing in lysosomes, mitochondria, or the ER (Figure S31). Additionally, fusion events between droplets were observed, a typical characteristic of LLPS (Figure 3c). In contrast, FITC‐PCNSs exhibited no such fusion behavior after cellular internalization in a short time (Figure S32).

**FIGURE 3:**
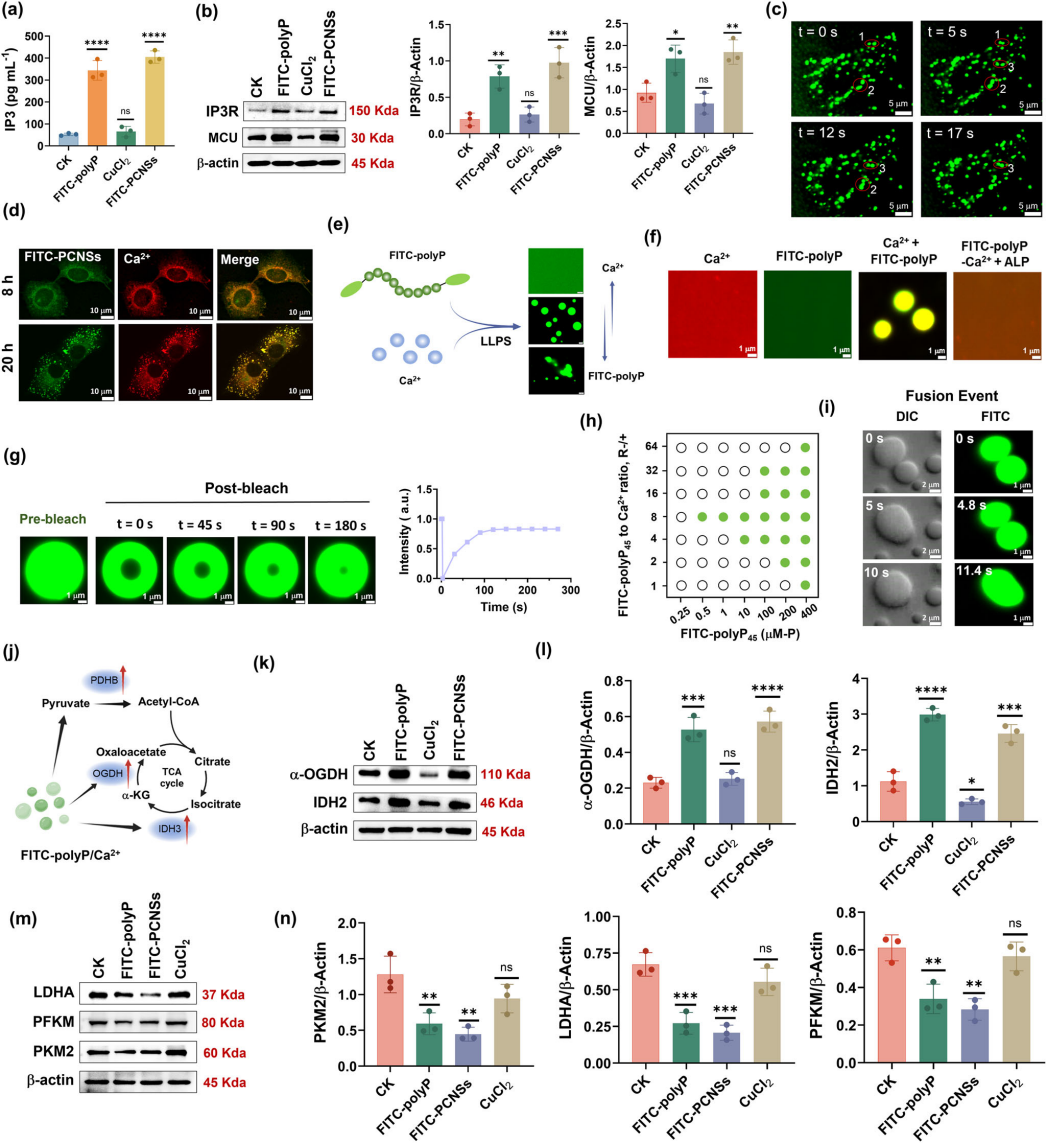
FITC‐polyP induces Ca^2+^ to undergo LLPS. (a) IP3 levels in culture medium after treatment with FITC‐polyP, CuCl_2_, and FITC‐PCNSs. (b) Western blot analysis of IP3R and MCU expression levels after FITC‐polyP, CuCl_2_, and FITC‐PCNSs treatment. (c) Images of the fusion of FITC‐polyP/Ca^2+^ condensates intracellularly. (d) Fluorescence co‐localization of intracellular FITC‐polyP condensates and Ca^2+^ following treatment with FITC‐PCNSs. (e) Schematic illustration of FITC‐polyP and Ca^2+^ LLPS and (f) CLSM images (Pi concentration was 400 µm, R_‐/+_ = 16). (g) CLSM images of droplets before and after partial photobleaching and fluorescence recovery curves. (h) Phase distribution of Ca^2+^ driven by heterogeneous FITC‐polyP, concerning FITC‐polyP concentration and the net charge ratio of FITC‐polyP to Ca^2+^, R_‐/+_. Solid green circles indicate FITC‐polyP concentrations for which spherical Ca^2+^ droplets were observed, while empty black circles represent cases where no coacervate droplets were observed. (i) Fusion of two FITC‐polyP/Ca^2+^ condensates. (j) Schematic illustration of the mitochondrial TCA cycle associated dehydrogenases activated by FITC‐polyP/Ca^2+^ condensates. (k,l) The key proteins in the TCA cycle, including α‐OGDH and IDH2 expression levels after FITC‐polyP, CuCl_2_, and FITC‐PCNSs treatment. (m,n) The key proteins in the glycolytic process, including LDHA, PFKM, and PKM2 expression levels after FITC‐polyP, CuCl_2_, and FITC‐PCNSs treatment. The concentrations of all materials in Figure a–c are: FITC‐polyP (0.057 µm, calculated), CuCl_2_ (100 µm), and FITC‐PCNSs (25 µg mL^−1^). All Data are displayed as mean ± standard deviation (*n* = 3). ^*^p < 0.05, ^**^p < 0.01, ^***^p < 0.001, and ^****^ *p* < 0.0001, analyzed by One‐Way ANOVA. “ns” indicates no significant difference.

Building on previous findings by Müller et al. regarding the formation of polyP/Ca^2+^ condensates [40], and considering the observed condensate fusion events alongside the potent divalent cation‐chelating capacity of FITC‐polyP, we hypothesize that these structures assemble via electrostatically mediated LLPS between FITC‐polyP and Ca^2+^. To confirm this, we first examined the extracellular LLPS behavior of FITC‐polyP in the presence of Ca^2+^. Upon mixing, the fluorescence signals of FITC‐polyP and Ca^2+^ were highly colocalized and exhibited a distinct tendency to form micron‐sized droplets, accompanied by significant turbidity changes (Figure 3e,f; Figure S33). Notably, this turbidity profile was highly consistent with that observed during the phase separation of unlabeled polyP with Ca^2+^, demonstrating that FITC conjugation does not appreciably perturb the phase separation behavior of polyP (Figure S34). Fluorescence recovery after photobleaching (FRAP) further revealed rapid molecular exchange within the droplets, a hallmark of LLPS (Figure 3g). A phase diagram constructed based on the negative‐to‐positive charge ratio (R_‐/+_) of FITC‐polyP to Ca^2+^ delineated the critical R_‐/+_ thresholds required for LLPS initiation at various FITC‐polyP concentrations (Figure 3h,i). Furthermore, increasing the ionic strength by raising the NaCl concentration from 150 mM to 1.0 M led to the progressive dissolution of the droplets. This result confirms that condensate formation is driven by reversible electrostatic interactions rather than irreversible precipitation (Figure S35). Next, we investigated whether the intracellular concentrations of FITC‐polyP released by FITC‐PCNSs after 12 h of treatment and endogenous Ca^2+^ could support LLPS. Intracellular FITC‐polyP concentrations reached 0.0276 µm after 12 h of FITC‐PCNSs treatment (Figure S36). Given this physiological concentration and the phase diagram (R_‐/+_ = 8), the Ca^2+^ concentration required to induce LLPS under these conditions was determined to be 0.078 µm (78 nm). This concentration lies within the physiologically relevant Ca^2+^ range reported in the literature [41, 42]. Furthermore, incubating the two components for 4 h at room temperature resulted in droplet formation in vitro (Figure S37). These results demonstrate that physiological Ca^2+^ concentrations could effectively induce FITC‐polyP phase separation. Combined with the experimental evidence of FITC‐polyP/Ca^2+^ LLPS occurring extracellularly, these findings further demonstrate that the observed condensates from Figure 3d are formed through LLPS between FITC‐polyP and Ca^2+^.

Based on the proteomic results, we further analyzed key proteins involved in the glycolytic and mitochondrial metabolic pathways using Western blot (WB) and RT‐PCR. Specifically, key TCA cycle dehydrogenases (α‐OGDH and IDH2) were markedly upregulated, whereas essential glycolytic enzymes (PFK, PKM2, and LDHA) were downregulated (Figure 3j–n; Figure S38). Given that Ca^2+^ is a central driver of mitochondrial respiration [37, 38, 39, 43], we reasoned that FITC‐polyP reprograms mitochondrial metabolism by triggering Ca^2+^‐mediated LLPS to reconfigure intracellular Ca^2+^ signaling networks. To systematically dissect the underlying molecular mechanism, we investigated the interplay between Ca^2+^ mobilization and condensate assembly. First, pharmacological blockade of ER Ca^2+^ release using the IP3R inhibitor 2‐Aminoethyl diphenylborinate (2‐APB) completely abolished the formation of FITC‐polyP/Ca^2+^ condensates, thereby preventing the downstream activation of the MCU and Ca^2+^‐responsive metabolic enzymes IDH2 and α‐OGDH (Figure 4a,b; Figure S39). This indicates that IP3R‐mediated Ca^2+^ efflux serves as the indispensable trigger for condensate assembly. Second, FITC‐PCNSs treatment significantly upregulated the Nudix hydrolase 3 (Nudt3), which exhibits polyphosphatase activity known to hydrolyze intracellular polyP during polyP accumulation [44]. Nudt3 expression increased 2.57‐fold in cells treated with FITC‐PCNSs for 24 h compared to controls (Figure 4c,d). Nudt3 progressively hydrolyzes polyP within the condensates, enabling a sustained, slow release of Ca^2+^. Genetic silencing of Nudt3 similarly blunted MCU activation and downstream dehydrogenase expression, establishing Nudt3 as the enzymatic engine that converts static condensates into dynamic “micro‐calcium depots” (Figure 4e,f; Figure S40). Finally, siRNA‐mediated knockdown of MCU abrogated the activation of Ca^2+^‐sensitive TCA cycle dehydrogenases α‐OGDH and IDH2, directly confirming MCU as the central gateway mediating mitochondrial Ca^2+^ influx and subsequent metabolic reprogramming (Figure 4g–j). Together, these findings demonstrate that FITC‐polyP/Ca^2+^ condensates function as intracellular “micro‐calcium depots” that precisely orchestrate Ca^2+^ signaling to drive tumor metabolic reprogramming toward OXPHOS.

**FIGURE 4:**
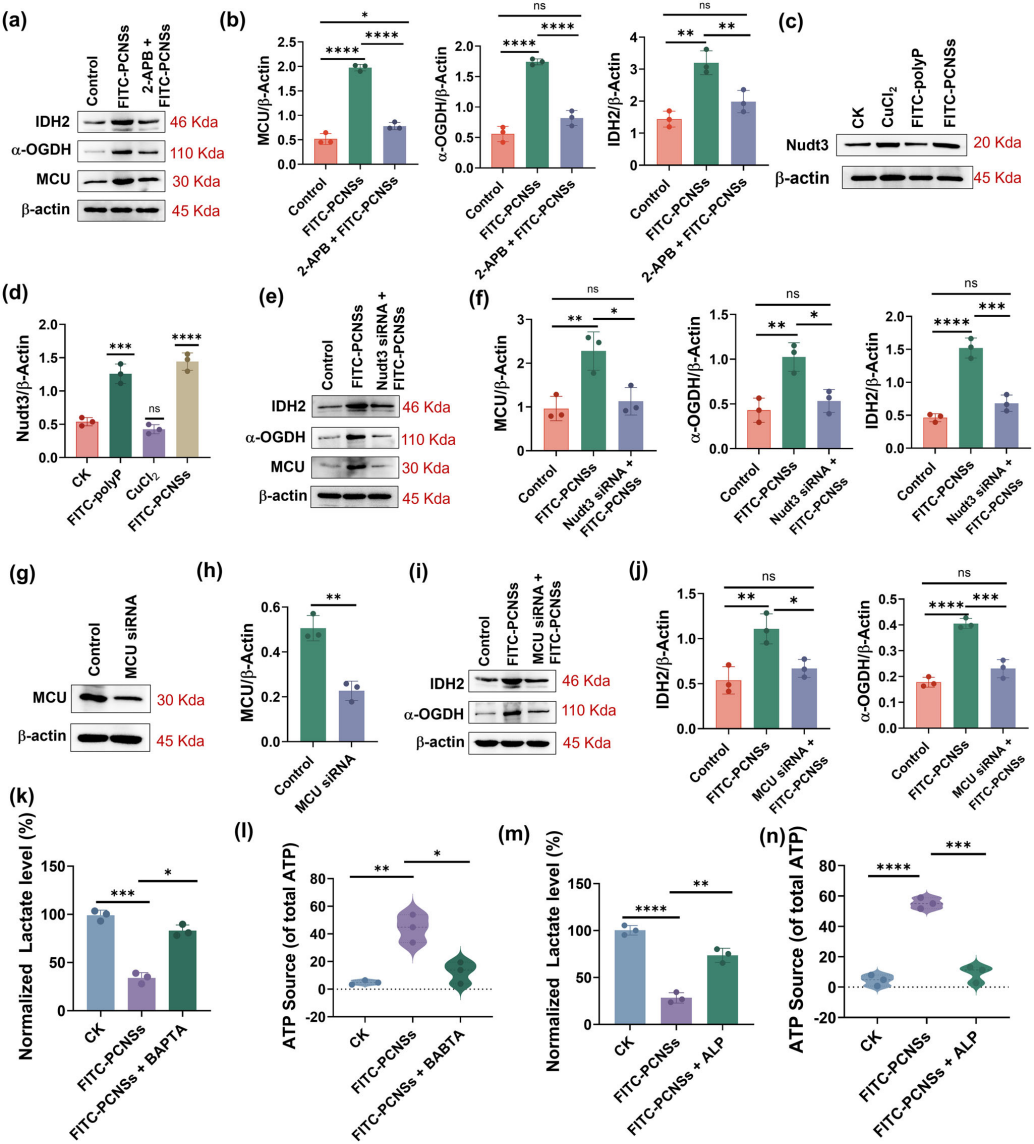
Mechanistic studies on the regulation of cellular metabolism by FITC‐polyP/Ca^2+^ condensates. (a, b) Expression of MCU, α‐OGDH, and IDH2 in cells treated with FITC‐PCNSs with or without 2‐APB pretreatment. (c, d) Nudt3 expression levels following treatment with FITC‐polyP, CuCl_2,_ or FITC‐PCNSs. (e, f) Expression levels of MCU, α‐OGDH, and IDH2 upon siRNA‐mediated Nudt3 knockdown. (g–j) Expression of MCU (g, h), as well as α‐OGDH and IDH2 (i, j) following siRNA‐mediated MCU knockdown. Normalized lactate level (k) and ATP production via OXPHOS (l) in cells pretreated with BAPTA/AM (20 µm), followed by treatment with FITC‐PCNSs. Normalized lactate level (m) and ATP production via OXPHOS (n) after treatment with FITC‐PCNSs followed by treatment with ALP (50 µg mL^−1^). The concentrations of all materials in Figure 4a,b,d,e,f,g are: FITC‐polyP (0.057 µm, calculated), CuCl_2_ (100 µm), and FITC‐PCNSs (25 µg mL^−1^). All Data are displayed as mean ± standard deviation (*n* = 3). ^*^p < 0.05, ^**^p < 0.01, ^***^p < 0.001, and ^****^ *p* < 0.0001; “ns” indicates no significant difference; data in Figure 4b,d,f,j,k,l,m,n were analyzed by One‐Way ANOVA; data in Figure 4h were analyzed by Student's *t*‐test.

To directly confirm the role of FITC‐polyP/Ca^2+^ condensates in mitochondrial activation, we disrupted condensate assembly using the Ca^2+^ chelator1,2‐Bis(2‐aminophenoxy)ethane‐N, N, N’, N’‐tetraacetic acid (BAPTA/AM) or the polyP‐degrading enzyme alkaline phosphatase (ALP). BAPTA/AM pretreatment prevented condensate formation and completely abolished the metabolic transition induced by FITC‐PCNSs (Figure 4k,l; Figure S41). Similarly, ALP treatment rapidly degraded intracellular condensates, reversing the metabolic shift by elevating lactate levels and reducing mitochondrial ATP production (Figure 4m,n; Figure S42 and S43). Together, these loss‐of‐function results directly establish FITC‐polyP/Ca^2+^ condensates as an indispensable molecular switch driving the metabolic transition from glycolysis to OXPHOS in tumor cells.

### 2.4 Metabolic Reprogramming Sensitizes Tumor Cells to Cuproptosis While Reducing Lactate Secretion to Alleviate Immunosuppression

Given that cuproptosis depends strictly on mitochondrial respiration, with respiration‐dependent cancer cells being up to 1,000‐fold more susceptible than glycolytic ones [45, 46, 47, 48], we investigated whether our metabolic remodeling sensitizes tumor cells to cuproptosis (Figure 5a). Following 24 h of FITC‐PCNSs treatment, cytosolic and mitochondrial Cu^2+^ levels reached 6.98 and 2.59 µg per 10^7^ cells, respectively, which confirmed targeted mitochondrial copper translocation (Figure S44). The half‐maximal inhibitory concentration (IC_50_) value of FITC‐PCNSs in 4T1 tumor cells was determined to be 23.55 µg mL^−1^, which is slightly lower than those observed in the aforementioned normal cells and virtually identical to the IC_50_ of unlabeled PCNSs against 4T1 cells (Figure 5b; Figure S45 and Table S2). This toxicity was substantially rescued by the cuproptosis inhibitor tetrathiomolybdate, validating cuproptosis induction (Figure 5c,d). Conversely, the OXPHOS inhibitor oligomycin significantly attenuated Cu‐elesclomol (Cu‐E; elesclomol is a potent copper ionophore that promotes cuproptosis) induced cytotoxicity, directly confirming that cuproptosis execution relies on active mitochondrial respiration (Figure 5e).

**FIGURE 5:**
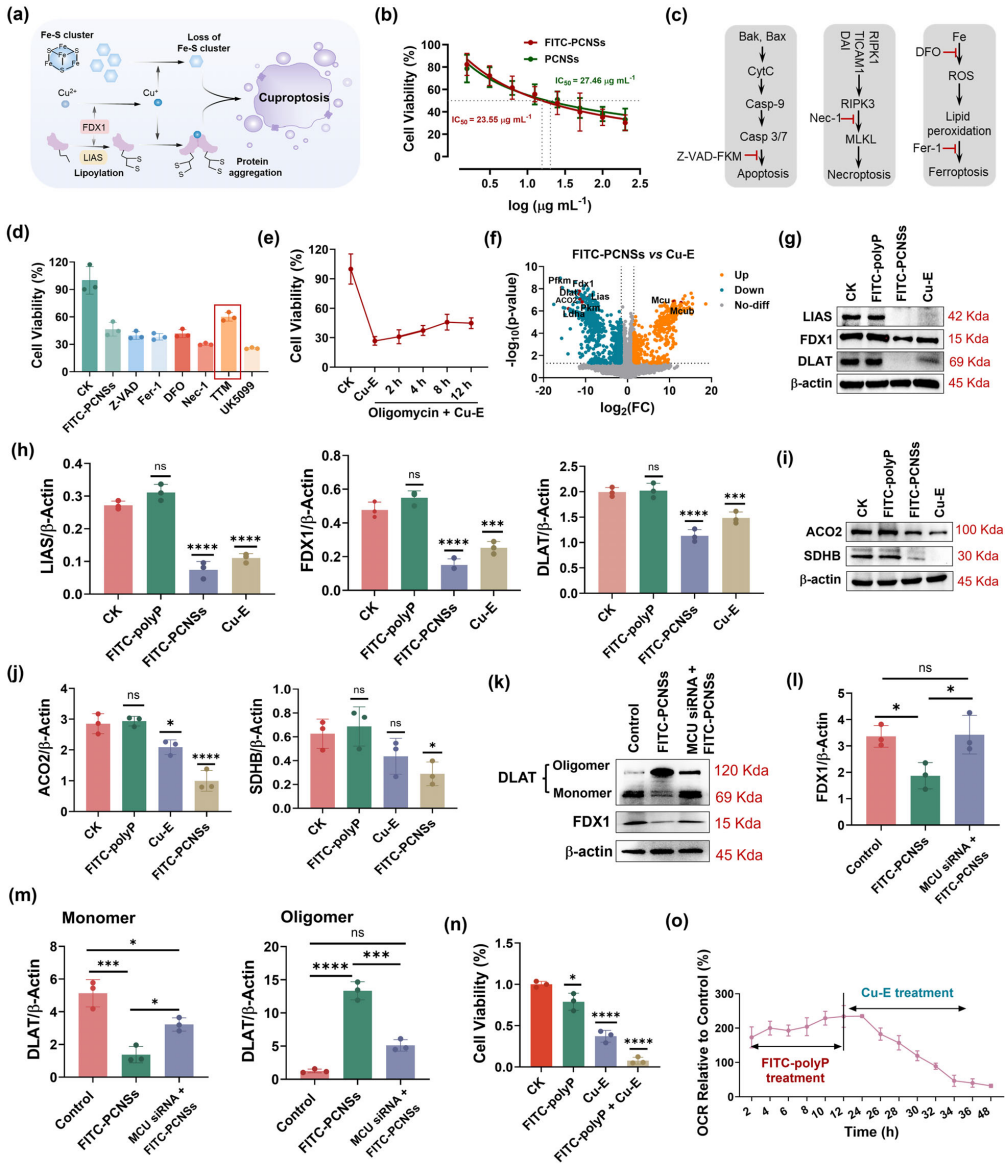
Metabolic reprogramming increases the susceptibility of tumor cells to cuproptosis. (a) Schematic illustration of the FITC‐PCNSs‐induced cuproptosis. (b) IC_50_ values of FITC‐PCNSs and PCNSs against 4T1 cells. (c) Schematic diagram of apoptosis, necroptosis, and ferroptosis. Inhibited pathways are marked in red. (d) Viability of 4T1 cells cultured with FITC‐PCNSs (25 µg mL^−1^) and different cell death inhibitors. (e) The cell viability of 4T1 cells was first treated with Cu‐E for 12 h and then treated with oligomycin (1.25 µm) for different periods. (f) Volcano plot of differentially expressed proteins in FITC‐PCNS‐treated 4T1 cells compared with Cu‐E‐treated cells. (g, h) WB analysis of the key proteins in cuproptosis, including LIAS, FDX1, and DLAT expression levels after FITC‐polyP, CuCl_2_, and FITC‐PCNSs treatment. (i, j) WB analysis of Fe‐S cluster proteins ACO2 and SDHB expression levels after FITC‐polyP, CuCl_2_, and FITC‐PCNSs treatment. (k–m) Expression levels of FDX1 and DLAT (oligomer and monomer) following siRNA‐mediated MCU knockdown. (n) The cell viability of 4T1 cells treated with FITC‐polyP, Cu‐E, and FITC‐polyP + Cu‐E for 24 h. (o) OCR of 4T1 cells, which were first treated with FITC‐polyP for 24 h and then were further exposed to Cu‐E for varying durations. The concentrations of all materials in Figure 5g,i,k are: FITC‐polyP (0.057 µm, calculated), CuCl_2_ (100 µm), and FITC‐PCNSs (25 µg mL^−1^). All Data are displayed as mean ± standard deviation (*n* = 3). ^*^p < 0.05, ^***^p < 0.001, and ^****^ *p* < 0.0001, analyzed by One‐Way ANOVA. “ns” indicates no significant difference.

To investigate whether FITC‐polyP induced metabolic reprogramming sensitizes tumor cells to copper‐induced cuproptosis, we first compared the proteomic profiles of 4T1 cells treated with FITC‐PCNSs versus Cu‐E. Key cuproptosis‐associated proteins, including ferredoxin 1 (FDX1), S‐acetyltransferase monomer (DLAT), and lipoic acid synthetase (LIAS), displayed markedly enhanced downregulation in the FITC‐PCNSs group (Figure 5f), a trend further validated by WB and RT‐PCR analyses (Figure 5g–j; Figure S46). We performed siRNA‐mediated silencing of FDX1 in 4T1 cells before FITC‐PCNSs exposure. Genetic depletion of FDX1 significantly conferred cell resistance to FITC‐PCNSs cytotoxicity, confirming that FITC‐PCNSs induce canonical cuproptosis (Figure S47). Crucially, attenuation of mitochondrial metabolism via siRNA‐mediated knockdown of MCU diminished the expression of TCA cycle dehydrogenases (α‐OGDH and IDH2), which concomitantly desensitized cells to cuproptosis (Figure 4g–j,k–m). Consistently, pharmacological inhibition of OXPHOS using oligomycin following FITC‐PCNSs treatment rescued the downregulation of cuproptosis‐related proteins (Figure S48). Most notably, sequential treatment with FITC‐polyP for 24 h prior to Cu‐E exposure resulted in a dramatic decline in cell viability and OCR compared to Cu‐E monotherapy (Figure 5n,o). Collectively, these findings establish that FITC‐polyP potentiates cuproptosis sensitivity in 4T1 cells by boosting mitochondrial metabolic flux.

### 2.5 Metabolic Reprogramming‐Based Therapeutic Approach Shows Excellent Antitumor Efficacy and Biosafety Validation In Vivo

Encouraged by the in vitro results, we further evaluated the in vivo antitumor efficacy of FITC‐PCNSs using a 4T1 tumor‐bearing mouse model. After 14 days of treatment with saline, FITC‐polyP, FITC‐PCNSs, or Cu‐E, the body weights of the mice remained stable, and the FITC‐PCNSs group exhibited a high survival rate of 75%, indicating minimal systemic toxicity at the administered dose (Figure 6a,d,f). Remarkably, FITC‐PCNSs demonstrated significant antitumor activity, achieving a tumor inhibition rate of 72.07%, as evidenced by marked reductions in tumor volume and weight (Figure 6b,c,g). Histopathological analysis via hematoxylin and eosin (H&E) staining revealed extensive tumor cell death, while immunofluorescence studies showed downregulation of FDX1, DLAT, and LIAS, confirming cuproptosis in tumor tissue (Figure 6e; Figures S49 and S50). Furthermore, we quantified the Cu levels in blood samples collected at multiple time points post‐administration. This analysis revealed a blood circulation half‐life of approximately 3.76 h (Figure 6h). Subsequently, analysis of Cu accumulation in tumor tissue and major organs showed that its concentration peaked at 24 h and was then gradually metabolized and cleared over time (Figure 6i; Figure S51). The tumor‐targeted delivery achieved in vivo relies predominantly on passive accumulation, commonly associated with the enhanced permeability and retention (EPR) effect [49, 50]. This process is highly favored by the rationally optimized size and the slight positive surface charge of the FITC‐PCNSs, facilitating their preferential enrichment at the tumor site. Importantly, routine hematological and biochemical analyses revealed no observable abnormalities, and H&E staining of major organs showed no noticeable pathological damage, supporting the favorable short‐term in vivo tolerability of FITC‐PCNSs (Figures S52–S54). To elucidate the metabolic regulatory effects of FITC‐PCNSs on tumors in vivo, we analyzed lactate secretion in the tumor microenvironment (TME) and the expression of glycolysis‐related proteins. Compared with the saline group, the FITC‐PCNSs‐treated group exhibited significantly reduced lactate levels and downregulated expression of PKM2 and LDHA (Figure 6j; Figure S55). These results highlight the ability of FITC‐PCNSs to effectively remodel tumor metabolism in vivo. To further validate the in vivo regulation of tumor metabolism by FITC‐polyP/Ca^2+^ condensates, we employed the specific MCU inhibitor, MCU‐i4. In 4T1 tumor‐bearing mice co‐treated with FITC‐PCNSs and MCU‐i4, blocking mitochondrial Ca^2+^ influx significantly attenuated the downstream effects of LLPS‐mediated signaling. Consequently, MCU‐i4 reversed the FITC‐PCNSs‐induced inhibition of glycolysis, restoring lactate release within the tumor microenvironment. This blunted metabolic activation subsequently rescued tumor cells from cuproptosis, ultimately compromising the overall in vivo anti‐tumor efficacy of the nanoplatform (Figure S56). Notably, the antitumor efficacy of FITC‐PCNSs was superior to that of Cu‐E, demonstrating that FITC‐polyP‐induced metabolic remodeling enhances susceptibility to cuproptosis at the mouse level (Figure 6b,c,g,e).

**FIGURE 6:**
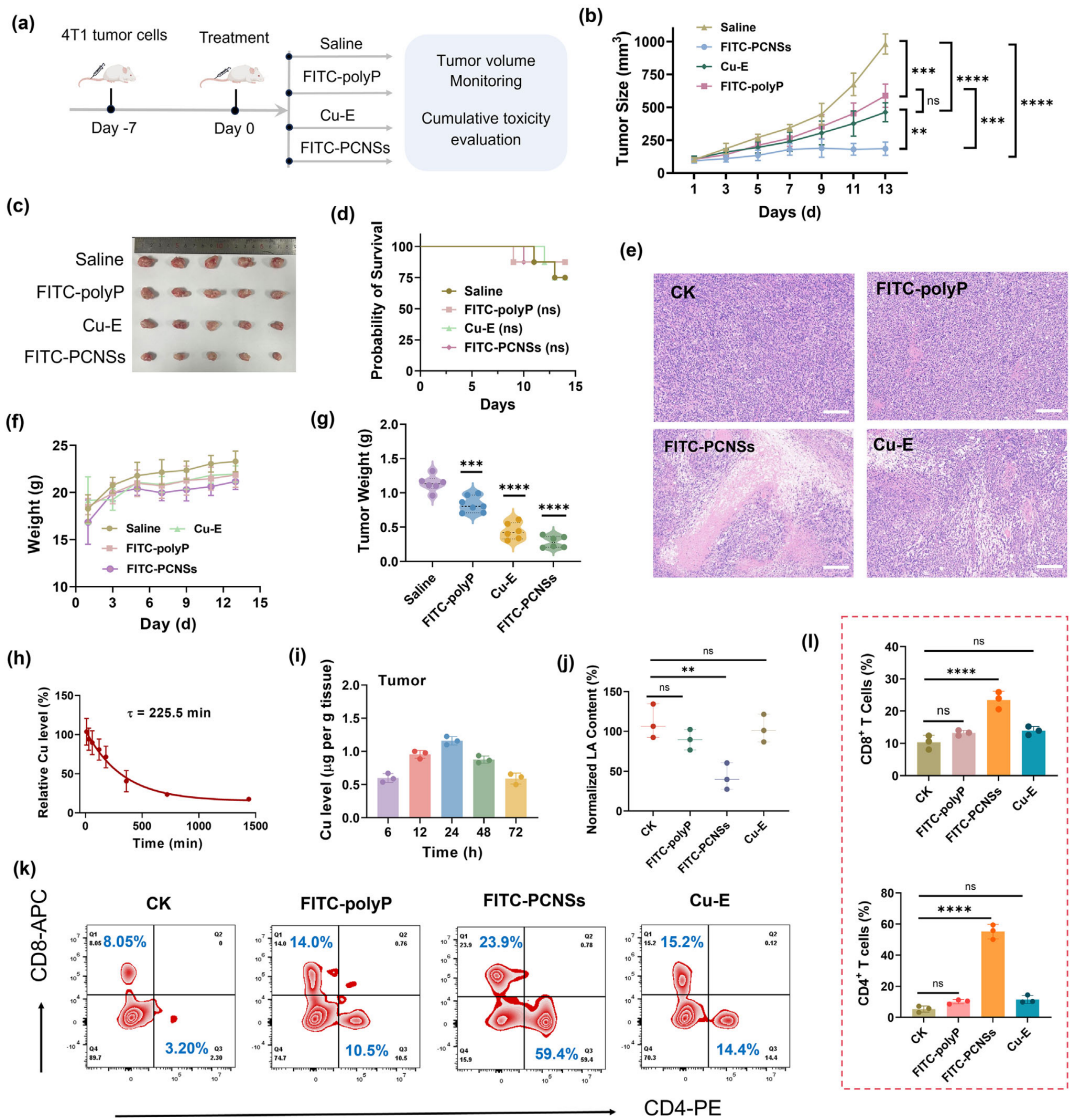
Assessment of metabolic reprogramming and anti‐tumor effects of FITC‐PCNSs in vivo. (a) Schematic illustration of the therapeutic process. FITC‐polyP and FITC‐PCNSs groups received intravenous injections at phosphorus‐equivalent doses of 15 mg P/kg, while FITC‐PCNSs and Cu‐E cohorts were administered copper‐equivalent doses of 10 mg Cu/kg via the same route. (b) Tumor size determination after the indicated treatments. (c) Representative tumor images after 14 days of FITC‐polyP, Cu‐E, and FITC‐PCNSs treatment in BALB/c mice bearing 4T1 tumors (*n* = 5). (d) Kaplan‐Meier survival curves of tumor‐bearing mice. 4T1 tumor‐bearing mice were randomly divided into 4 groups and treated with Saline, PolyP, PolyP‐Cu, or Cu‐E. Survival data are presented as a Kaplan‐Meier plot. Statistical significance between individual treatment groups and the control group was determined by pairwise log‐rank (Mantel‐Cox) tests. The Bonferroni correction was applied to adjust for multiple comparisons. (e) Representative micrographs of tumor tissues after H&E staining. Scale bar: 50 µm. (f) The body weights of the mice after the indicated treatments. (g) Weights of tumor tissues in mice after 14 days of treatment. (h) Blood half‐life of FITC‐PCNSs based on ICP‐MS detection of copper content. (i) Distribution of Cu in tumor tissue after administration. (j) Emission of lactate in the TME after 14 days of treatment. The flow cytometry plots of CD4^+^ T cells and CD8^+^ T cells in tumors with treatments (gated on CD3^+^ cells) (k) and statistical analysis of CD8^+^ T cells and CD4^+^ T cells in the TME (l). All Data are displayed as mean ± standard deviation (*n* = 3). ^**^p < 0.01, ^***^p < 0.001, and ^****^ *p* < 0.0001; “ns” indicates no significant difference; data in Figure 6b were analyzed by Two‐Way ANOVA; data in Figures 6g,j,l were analyzed by One‐Way ANOVA.

We subsequently investigated the immune cell dynamics within the tumor TME following 14 days of treatment with FITC‐PCNSs. Flow cytometry analysis of the tumor revealed that FITC‐PCNSs induced an increase in the number of CD4^+^ and CD8^+^ T cells in the tumor (Figure 6k,l). We next detected the subsequent effector tumor‐specific CD8^+^ T cells and Treg cells among the CD4^+^ T cells. Our results showed that FITC‐PCNSs treatment significantly promoted an increase in the number of CD8^+^/IFN‐γ^+^ T cells and a decrease in the number of CD4^+^/Foxp3^+^ Treg cells in the TME (Figures S57 and S58). In contrast, FITC‐polyP alone did not enhance cytokine production, likely due to its high negative charge density, which may hinder cellular uptake. Overall, these findings indicate that FITC‐PCNSs mediated tumor metabolic reprogramming and subsequent cuproptosis co‐occur with favorable immune remodeling in the TME, characterized by reduced lactate levels and enhanced local T‐cell accumulation. It should be noted that while FITC‐PCNSs treatment leads to reduced lactate secretion and elevated CD8^+^ T‐cell infiltration in vivo, the current immunological data remain correlative. Additional factors, such as copper‐induced tissue inflammation, immunogenic debris from cuproptosis, or altered tumor vascular permeability, may also contribute to the observed T‐cell accumulation. Definitive proof of immune‐dependent tumor control, such as CD8^+^ T‐cell depletion studies or experiments in immunodeficient mouse models, warrants future investigation.

## 3 Conclusion

In this study, we developed a series of highly biocompatible polyP‐based nanoplatforms via terminal FITC functionalization of the biopolymer polyP. The resulting nanosystem enables programmed polyP release under physiological conditions while effectively mediating Ca^2+^‐induced LLPS. Serving as “micro calcium depots,” the assembled FITC‐polyP/Ca^2+^ condensates markedly enhance mitochondrial Ca^2+^ influx through MCU activation. This influx drives metabolic remodeling by upregulating key rate‐limiting TCA cycle enzymes (IDH2 and α‐OGDH). Consequently, these events elevate OXPHOS flux and dictate a metabolic transition from glycolysis to mitochondrial oxidative metabolism. Crucially, the complete abolition of this metabolic shift upon Ca^2+^ chelation with BAPTA/AM or enzymatic clearance with ALP underscores that polyP/Ca^2+^ phase condensation acts as a pivotal molecular switch directing cellular metabolic fates.

Through this phase‐separation‐driven metabolic rewiring, the tumor cells enter a “metabolically sensitized” state primed for cuproptosis. The concurrently released Cu^2+^ is reduced to Cu^+^ by FDX1 and directly binds DLAT, thereby executing a self‐sensitizing cuproptosis. In tumor‐bearing mouse models, the nanoformulation successfully induced metabolic remodeling and cuproptosis in vivo, driving marked tumor regression along with favorable remodeling of the tumor microenvironment. Although the nanoplatform exhibited acceptable short‐term biocompatibility without acute systemic lesions in the tested 4T1 syngeneic model, several translational limitations should be acknowledged. First, the present safety profile was established over a short observation window within a single animal model. Given the potential metabolic risks associated with repeated intravenous copper dosing, comprehensive long‐term chronic toxicity, systemic pharmacokinetic clearance, and biodistribution analyses across diverse preclinical models remain imperative for future clinical translation. Furthermore, because the current nanoassembly relies primarily on passive EPR effect‐mediated accumulation, engineering active target‐homing ligands onto the surface of PCNSs represents a critical next step to minimize off‐target exposure and enhance delivery efficiency.

Finally, it should be emphasized that FITC conjugation was employed exclusively as an analytical imaging tracer for intracellular trafficking and biodistribution monitoring. Because unlabeled PCNSs exhibit physicochemical characteristics, phase separation kinetics, and cytotoxic potency equivalent to those of FITC‐PCNSs, the pristine, unlabeled PCNSs formulation represents the authentic, clinically translatable therapeutic candidate, mitigating the potential regulatory and immunological liabilities associated with synthetic fluorophores.

## 4 Methods

### 4.1 Preparation of FITC‐PCNSs

A solution of FITC‐polyP (0.032 mmol, 225 mg) and Urea (0.75 mmol, 45.45 mg) in ddH_2_O (15 mL, pH 8) was prepared. Then, the system was ultrasonically dispersed for 30 min. A solution of CuSO_4_ (10.00 mmol, 1.596 g) in ddH_2_O (15 mL, pH 6.5) was stirred for 30 min at room temperature. After mixing the above solutions, the mixture was transferred to a Teflon reactor and reacted at 60°C for 12 h. The resulting blue product was then obtained by centrifugation and washed with ddH_2_O. The samples were lyophilized for 2 days.

### 4.2 Evaluation of Glycolytic Capacity

Cells were seeded in 24‐well plates at 2.5 × 10^4^ cells per well and cultured for 24 h. Control, FITC‐PCNSs, CuCl_2_, and FITC‐polyP treatment groups were established, with each treatment group further divided into oligomycin (+) and oligomycin (‐) subgroups. The culture medium was removed, and the experimental groups were treated with FITC‐PCNSs (25 µg mL^−1^), CuCl_2_ (100 µm), and FITC‐polyP for 24 h (FITC‐polyP was introduced into the cells via transfection, 0.057 µm, calculated). Following treatment, the medium was removed again, and the cells were gently washed three times with PBS. Subsequently, 200 µL of culture medium containing 1.25 µm oligomycin was added to the oligomycin (+) subgroup, while the oligomycin (−) subgroup received medium without oligomycin. The cells were incubated for an additional 3 h; 100 µL of culture medium was collected from each well, and the lactate in the medium was measured.

### 4.3 Assessment of Lactate Content

Lactate levels were determined by a Glycolysis/OXPHOS Assay Kit (DOJINDO LABORATORIES, G270). 20 µL of the sample was added to each well of a 96‐well plate, followed by the addition of 80 µL of lactate working solution to each well. The mixture was incubated for 30 min, after which the absorbance was recorded at 450 nm.

### 4.4 Assessment of OCR

OCR was determined by the Extracellular OCR Plate Assay Kit (DOJINDO LABORATORIES, E‐297). Cells were seeded in 96‐well black plates at 3 × 10^4^ cells per well and cultured for 24 h. Blank1, Blank2, Blank3, control, and sample groups were established accordingly. Following overnight incubation, the wells designated for the sample treatment were exposed to FITC‐polyP (0.057 µm, calculated), FITC‐PCNSs (25 µg mL^−1^), and CuCl_2_ (100 µm) for 24 h. Subsequently, the medium was removed, and 100 µL of fresh medium was added to the wells of Blank1 and Blank3. In contrast, 100 µL of a working solution diluted from the oxygen probe was introduced into the wells of Blank2, control, and sample groups. After an additional incubation of 30 min, one drop of mineral oil was added to each well. The fluorescence intensity (E_X_ = 500 nm, E_m_ = 650 nm) was continuously read and detected at 10 min intervals for 200 min, and OCR was calculated.

### 4.5 Assessment of NAD^+^/NADH Ratio

NAD^+^/NADH was determined by the NAD/NADH Assay Kit‐WST (DOJINDO LABORATORIES, N509). Cells were seeded in 6‐well black plates at 3 × 10^5^ cells per well and cultured for 24 h. The treatment was continued for 24 h with FITC‐polyP (0.057 µm, calculated), FITC‐PCNSs (25 µg mL^−1^), and CuCl_2_ (100 µm), respectively. Subsequently, the cells from each treatment group were collected, and further procedures were performed according to the manufacturer's instructions provided in the kit.

### 4.6 Cell Viability Assay

Cells were seeded in 96‐well plates at 1 × 10^4^ cells per well and cultured for 24 h. After the cells were treated with different concentrations of FITC‐PCNSs for 24 h, 10 µL per well of CCK8 solution (KeyGEN, Nanjing, China) was added to the cells and incubated for an additional 4 h. Finally, OD_450_ was obtained with a microplate reader.

### 4.7 Western Blot Analysis

4T1 cells were seeded in 6‐well tissue culture plates at a density of 1.5 ×10^5^ cells mL^−1^ for 24 h. After treatment with FITC‐polyP (0.057 µm, calculated), FITC‐PCNSs (25 µg mL^−1^), and CuCl_2_ (100 µm) for 24 h, the cells were collected, and then the cell lysates were obtained with a Whole Cell Lysis Assay. The protein concentration was determined by the Super‐Bradford Protein Assay Kit (CWBiotech, Inc., Beijing, China). Briefly, the extracts were first separated by 10% SDS‒PAGE and transferred to a polyvinylidene difluoride membrane (Bio‐Rad, CA, USA). The membrane was blocked with 5% BSA in TBST at 25°C for 1 h and then incubated with antibodies at 4°C overnight. The expression of β‐actin was used as the internal standard. Primary antibodies against the following proteins were used: β‐actin (1:1000 for WB), PKM2 (1:1000 for WB), LDHA (1:1000 for WB), PFKM (1:1000 for WB), α‐OGDH (1:1000 for WB), IDH2 (1:1000 for WB), PDHB (1:1000 for WB), IP3R (1:1000 for WB), MCU (1:1000 for WB), LIAS (1:1000 for WB), FDX1 (1:1000 for WB), DLAT (1:1000 for WB), ACO2 (1:1000 for WB), SDHB (1:1000 for WB). The appropriate secondary antibodies (1:1000 for WB) were purchased from CST. For the experiments, different target proteins were analyzed on separate membranes using aliquots from the same batch of protein lysates, with identical sample order and equal protein loading. β‐Actin was analyzed in parallel on a separate membrane using the same set of lysates and served as a common loading reference for these parallel blots. For our earlier experiments, the transferred membrane was physically sectioned according to the expected molecular weights of the target proteins before antibody incubation. Therefore, a single antibody‐exposed full‐length membrane image does not exist for these experiments. We have provided all available original images of the corresponding membrane sections. For the additional Western blot data provided during the subsequent review, the membranes were left uncropped before antibody incubation, allowing us to provide full‐length blot images for these experiments (Figures S59–S73).

### 4.8 Phase Separation of the FITC‐polyP/Ca^2+^ System

Commercially available heterogeneous polyP45 (also named “sodium phosphate glass”, Sigma‐Aldrich) was dissolved in ddH_2_O to prepare 2× stock solutions at specified Pi concentrations (0.25 µm, 0.5 µm, 1 µm, 10 µm, 100 µm, 200 µm, and 400 µm Pi). Corresponding 2× Ca^2+^ stock solutions (concentrations ranging from 0.031 µm to 100 µm) were also prepared in this buffer. The Ca^2+^ concentrations were derived from the FITC‐polyP to Ca^2+^ net charge ratio (R_‐/+_), as detailed in Figure 4i. In vitro Ca^2+^ Phase separation was then performed by mixing equal volumes of FITC‐polyP and Ca^2+^ stock solution, after which the mixture was transferred into a Petri dish and incubated at room temperature till the imaging process was finished within 4 h. Confocal images were taken on an OLYMPUS SpinSR confocal laser scanning microscope equipped with multiple laser units, a live cell chamber, and a built‐in FRAP function. Images were captured using a UPLXAPO 100 × oil immersion objective and two channels of a cooled GaAsP PMT detector. Image analysis was performed with the manufacturer's software, CellSens.

### 4.9 Fluorescence Recovery after Photobleaching (FRAP)

For bleaching performed inside droplets, circular regions (1.5–2.0 mm diameter) in the centre of droplets (6.0–10.0 mm diameter) were selected as regions of interest (ROI). FITC was excited at 488 nm, and emission signals were collected in the range 500–520 nm. Ten images at attenuated laser intensity (20% intensity) were taken before photobleaching. Photobleaching was performed using a single pulse of an argon laser (405 nm) at 50% intensity for FITC (25 frames) through a round region of interest ROI. The laser was then switched back to attenuated intensity, and the recovery images were recorded for 100 s (200 frames). The time interval of each frame is 0.5 s. The recorded fluorescence intensities from the bleached ROI were then corrected for photofading and normalized using the FRAP function module of CellSens. Data fitting was performed with an exponential function f(t) = A [1 – exp(‐t/t)] using GraphPad Prism.

### 4.10 Coacervate Microdroplet Fusion

The fusion tests between the coacervate microdroplets in aqueous solution were conducted by mixing the FITC‐polyP and Ca^2+^ at a ratio of R_‐/+_ = 16. The fusion processes of the polyP/Ca^2+^ coacervate microdroplets were monitored under bright‐field and 488 nm fluorescence channels in an OLYMPUS SpinSR confocal laser scanning microscope, respectively.

### 4.11 Evaluation of the In Vivo Therapeutic Efficacy of FITC‐PCNSs

All animal experiments were performed following the National Institutes of Health animal care guidelines. Animal protocols were approved by the Institutional Animal Care and Use Committee (Nanjing University, IACUC‐D2202146). To establish the tumor‐bearing mouse model, female BALB/c mice purchased from the Model Animal Research Center of Nanjing University (Jiangsu, China) were subcutaneously injected into the left foreleg armpit of each mouse with 10^6^ 4T1 cells in 100 µL of PBS. Therapeutic interventions commenced when tumor volumes reached approximately 100 mm^3^, administered via tail vein injection (saline, FITC‐polyP, FITC‐PCNSs, or Cu‐E) on days 0, 3, 6, 9, 12, and 14. Systemic delivery protocols were concurrently implemented: FITC‐polyP and FITC‐PCNSs groups received intravenous injections at phosphorus‐equivalent doses of 15 mg P/kg, while FITC‐PCNSs and Cu‐E cohorts were administered copper‐equivalent doses of 10 mg Cu/kg via the same route. Initially, the mice were randomly divided into 4 groups (n = 8 per group). Over the 14‐day treatment period, mice that died prior to the study endpoint due to tumor burden were excluded from the final endpoint analyses. On day 14, tumors from all surviving mice were excised and weighed to assess the therapeutic efficacy. The tumor size and weight of all groups were measured once every two days. Tumor size was determined by a Vernier caliper at frequent intervals, and the tumor volume (*V*) was calculated by the formula:

(1) $$V\left(mm^{3}\right)=\frac{L\times W^{2}}{2}$$

where *L* is the length of the tumor, and *W* is the width of the tumor. The mice were randomly divided into 4 groups (*n* = 8 per group). Moreover, the survival of tumor‐bearing mice treated with different modalities was monitored. On day 14, the mice were euthanized, and the tumor tissue was collected and fixed with 10% neutral buffered formalin. Then, the tumors were embedded in paraffin, processed into 100 µm sections, and stained with hematoxylin and eosin (H&E). The images were obtained by digital microscopy.

### 4.12 Tumor Metabolic Reprogramming and Cuproptosis Sensitization Assessment

At the end of treatment, tumor tissue was collected from each group for an immunofluorescence assay. Tumor tissues were fixed with 4% PFA and embedded in paraffin. The paraffin‐embedded samples were sectioned into 5 µm‐thick histologic slides, which were blocked for endogenous activity with 2% H_2_O_2_ and pre‐treated with Diva Decloaker buffer (Biocare Medical) for heat retrieval. The slides were washed with Tris‐buffered saline with 0.025% Tween 20 detergent (TBST) and then blocked by incubation in Biocare Medical Background Sniper in 2% bovine serum albumin/PBS solution for 10 min. After removing the blocking buffer, anti‐mouse monoclonal antibodies against PKM2 (1:50, Abcam), LDHA (1:50, Abcam), FDX1 (1:50, Abcam), DLAT (1:50, Abcam), and LIAS (1:50, Abcam) were applied to slides overnight at 4°C in the dark. After re‐staining with fluorescent secondary antibody and DAPI, the slices were imaged under a confocal laser scanning microscope (ZISSE). Lactate levels in tumour tissue fluid were measured using the Lactate Content Monitoring Kit (Solarbio).

### 4.13 In Vivo Toxicity of FITC‐PCNSs

Blood samples were collected from mice across treatment groups for comprehensive hematological profiling and serum biochemical analysis. Hematological parameters were quantified using a Photoelectric MEK‐8222K automated five‐class blood cell analyzer. Serum biochemical markers were assessed with commercially available assay kits (Nanjing Jiancheng Bioengineering Institute, China). Major organs (liver, spleen, kidney, heart, and lung) were harvested and subjected to standardized histopathological processing: fixation in 10% neutral buffered formalin, paraffin embedding, sectioning, H&E staining, and subsequent digital microscopic evaluation.

### 4.14 Immune Activation Detection of FITC‐PCNSs In Vivo

The mouse model was established as described above, and tumor tissues were harvested on day 14 post‐intervention. To maintain high cell viability for flow cytometry, fresh tumors from 3 randomly selected surviving mice per group (n = 3) were immediately harvested on day 14 and processed into single‐cell suspensions. Single‐cell suspensions underwent sequential immunophenotyping: initial staining with anti‐CD3 FITC, anti‐CD4 PE, and anti‐CD8 APC antibodies for T‐cell subset analysis via flow cytometry, followed by anti‐CD3 FITC/anti‐CD8a PE/anti‐IFN–*γ* APC triple staining to quantify tumor‐specific CD8^+^ effector T cells. Regulatory T cells (Tregs) were identified using anti‐CD4 PE and anti‐Foxp3 FITC dual labeling, supplemented with anti‐CD25 APC for splenic Treg validation. Concurrently, serum cytokines including IL‐6, IFN–*γ*, and TNF‐α were quantified using commercial ELISA kits.

### 4.15 Statistical Analysis

All quantitative data were processed without arbitrary exclusion or subjective transformation. Data normalization (e.g., percentage of control) was performed against untreated control groups. Exact sample sizes (n) for in vitro and in vivo cohorts are specified in the respective figure legends. All data are presented as the mean ± standard deviation (SD). Comparisons between two independent groups were performed using a two‐tailed Student's *t*‐test. One‐Way ANOVA was utilized for comparisons among three or more groups at a single time point, followed by Tukey's post hoc test for multiple pairwise comparisons or Dunnett's post hoc test for comparisons against a single control group. Tumor volumes were analyzed by Two‐Way ANOVA with Tukey's post hoc test. Cumulative survival curves were plotted using the Kaplan–Meier method and analyzed via the log‐rank (Mantel–Cox) test. Statistical significance was set at ^*^ *p* < 0.05, ^**^p < 0.01, ^***^p < 0.001, and ^****^p< 0.0001. “ns” indicates no significant difference. All analyses were performed using Prism version 10.0 (GraphPad, San Diego, USA).

## Author Contributions

**Jing Wang**: conceptualization, writing – review and editing, writing – original draft, methodology, formal analysis, investigation, data curation, visualization, software, validation. **Xinyang Zhao**: validation, methodology, writing – review and editing, investigation, data curation, software, writing – original draft. **Zifeng Huang**: investigation, methodology, formal analysis. **Jingyi Zhang**: methodology, formal analysis, data curation. **Yucheng Tao**: methodology, software. **Yong Huang**: funding acquisition, supervision. **Zijian Guo**: conceptualization, funding acquisition, supervision. **Lei Dong**: funding acquisition, supervision, project administration. **Xiuxiu Wang**: supervision, funding acquisition, project administration. **Jing Zhao**: funding acquisition, project administration, supervision, conceptualization, resources. **Wei Wei**: resources, supervision, funding acquisition, project administration.

## Conflicts of Interest

The authors declare no conflicts of interest.

## Supporting information




**Supporting File**: advs78368‐sup‐0001‐SuppMat.docx.

## Data Availability

The data that support the findings of this study are available from the corresponding author upon reasonable request.
