# Supplementary material for: Polyphosphate Copper Nanospheres Drive Mitochondrial Metabolic Reprogramming via Calcium Phase Separation for Self‐Sensitizing Cuproptosis
Source: Adv Sci (Weinh). 2026 Oct 22:e78368. Online ahead of print. doi: 10.1002/advs.78368 (PMC13690975; doi:10.1002/advs.78368)
Supplement: Supplementary file 1 [file ADVS-9999-e78368-s001.docx]

Supporting Information

Polyphosphate Copper Nanospheres Drive Mitochondrial Metabolic Reprogramming via Calcium Phase Separation for Self-Sensitizing Cuproptosis

Jing Wang, Xinyang Zhao, Jingyi Zhang, Zifeng Huang, Yucheng Tao, Yong Huang, Zijian Guo, Lei Dong,^*^ Xiuxiu Wang,^*^ Jing Zhao,^*^ and Wei Wei^*^

**Content**

[Experimental Section	3](#_Toc237442296)

[Reagents and cell lines.	3](#_Toc237442297)

[Synthesis of amine-labeled polyP-NH_2_.	3](#_Toc237442298)

[Preparation of FITC-labeled polyP (FITC-polyP).	4](#_Toc237442299)

[Preparation of FITC-PCNSs.	4](#_Toc237442300)

[Characterization of nanomaterials.	4](#_Toc237442301)

[Evaluation of glycolytic capacity.	5](#_Toc237442302)

[Assessment of lactate content.	5](#_Toc237442303)

[Intracellular transfection and content assessment of FITC-polyP.	5](#_Toc237442304)

[Assessment of OCR.	5](#_Toc237442305)

[Assessment of NAD^+^/NADH ratio.	6](#_Toc237442306)

[Cu^2+^ is released at different pH values.	6](#_Toc237442307)

[GPC analysis.	6](#_Toc237442308)

[^31^P NMR analysis.	^6^](#_Toc237442309)

[Cell viability assay.	6](#_Toc237442310)

[Western blot analysis.	6](#_Toc237442311)

[Quantitative RT-PCR.	7](#_Toc237442312)

[ICP-OES/MS Measurement Protocol.	8](#_Toc237442313)

[siRNA Transfection Analysis.	8](#_Toc237442314)

[*In Vitro* Cellular Uptake of FITC-PCNSs.	8](#_Toc237442315)

[Intracellular Ca^2+^ imaging.	9](#_Toc237442316)

[Phase separation of the FITC-polyP/Ca^2+^ system.	9](#_Toc237442317)

[Fluorescence recovery after photobleaching (FRAP).	9](#_Toc237442318)

[Coacervate microdroplet fusion.	9](#_Toc237442319)

[Evaluation of the *in vivo* therapeutic efficacy of FITC-PCNSs.	10](#_Toc237442320)

[Tumor metabolic reprogramming and cuproptosis sensitization assessment.	10](#_Toc237442321)

[*In vivo* toxicity of FITC-PCNSs.	11](#_Toc237442322)

[Immune activation detection of FITC-PCNSs *in vivo*.	11](#_Toc237442323)

[Statistics and reproducibility.	11](#_Toc237442324)

[Results	12](#_Toc237442325)

Experimental Section

Reagents and cell lines. Polyphosphate is composed of approximately 45 phosphate groups (polyP_45_) and is available commercially; we purchased it from Sigma. MnCl_2_ was purchased from Sigma (USA), and 2,2-(ethylenedioxy)bis-(ethylamine) was purchased from Aladdin. Urea was purchased from Mackli. Sodium dodecyl sulfate (SDS) was purchased from Aladdin. Fluorescein isothiocyanate (FITC) was obtained from Energy Chemical. The above reagents were used as received without any further purification. Human neuroblastoma cells (SH-SY5Y), mouse breast cancer cells (4T1), human embryonic lung fibroblast cells (MRC-5), human monocytic cells (THP-1), and human embryonic kidney cells (HEK-293T) were obtained from the Type Culture Collection of the Chinese Academy of Sciences (Shanghai, China). All cell lines were authenticated via STR typing by the Type Culture Collection of the Chinese Academy of Sciences and were confirmed to be free of cross-contamination and mycoplasma. Further details on the cell lines are provided in Table S1. SH-SY5Y cells were cultured in 50% MEM nutrient medium (MEM, Gibco, NY, USA) and 50% Ham's F-12 nutrient medium (Ham's F-12, Gibco, NY, USA); 4T1 cells were cultured in RPMI 1640 nutrient medium (1640, Gibco, NY, USA); MRC5 cells were cultured in MEM nutrient medium (MEM, Gibco, NY, USA); THP-1 cells were cultured in RPMI 1640 nutrient medium (1640, Gibco, NY, USA); HEK 293T cells were cultured in DMEM nutrient medium (DMEM, Gibco, NY, USA); All medium were added 10% fetal bovine serum (FBS, Gibco, NY, USA), 100 units mL^-1^ penicillin, and 50 units mL^-1^ streptomycin at 37°C in a CO_2_ incubator (95% relative humidity, 5% CO_2_).

**Table S1.** Details of cell lines used in this study.

| Cell lines | RRIDs | Identifier | Date of purchase |
| --- | --- | --- | --- |
| SH-SY5Y | CVCL_0019 | CSTR:19375.09.3101HUMSCSP5014 | 2023.04 |
| 4T1 | CVCL_0125 | CSTR:19375.09.3101MOUSCSP5056 | 2022.02 |
| MRC-5 | CVCL_0440 | CSTR:19375.09.3101HUMSCSP5040 | 2025.03 |
| THP-1 | CVCL_0006 | CSTR:19375.09.3101HUMSCSP567 | 2024.06 |
| HEK-293T | CVCL_0063 | CSTR:19375.09.3101HUMSCSP502 | 2024.07 |

Synthesis of amine-labeled polyP-NH_2_. PolyP-NH_2_ was synthesized following a modification of Wender’s protocol^[51]^. PolyP 45 was dissolved in 1 M MES buffer to a final concentration of 50 mM. 2,2’-(ethylenedioxy)bis(ethylamine) (20 equiv.) was added to the polyP solutions, followed by the addition of 1-ethyl-3-(3-dimethylaminopropyl)carbodiimide hydrochloride (EDAC) (20 equiv.). The pH of the mixture was adjusted to 6.5 using 1 M HCl. The reaction mixture was then heated to 60 °C for 2 h. Subsequently, the pH was raised to 10 with 1 M NaOH, and the crude product was purified by precipitation into 45 mL of ethanol, repeated three times. The resulting precipitate was collected by centrifugation (6 min at 1100 x g) and lyophilized to obtain a dry product. ^1^H NMR (400 MHz, D_2_O) δ 3.78 - 3.54 (m, 16H), 3.17 (t, J = 5.1 Hz, 4H), 3.06 (dt, J = 10.6, 5.6 Hz, 4H). ^31^P NMR (162 MHz, D_2_O) δ 2.82, -0.38, -0.49, -4.53, -4.64, -19.84, -19.93, -20.76, -21.19, -21.39, -21.54, -21.77, -22.34, -23.36. ^13^C NMR (101 MHz, D_2_O) δ 71.15, 71.06, 69.57, 69.36, 66.64, 40.85, 39.12.

Preparation of FITC-labeled polyP (FITC-polyP). PolyP-NH_2_ was dissolved in a 0.1 M carbonate buffer at pH 9.0 to a final concentration of 100 mM. FITC (5 equiv) was added to the solution, and the reaction mixture was stirred for 20 h at room temperature. The crude product was purified by precipitation into 45 mL of ethanol, repeated three times. The precipitate was collected by centrifugation at 1,100 x g for 15 minutes. The resulting material was then dissolved in alkaline water (pH 8.0) and further purified by preparative size exclusion chromatography with Sephadex 25. Finally, the samples were lyophilized for 48 h. ^1^H NMR (400 MHz, D_2_O) δ 7.89 – 7.78 (m, 0.16H), 7.46 – 7.39 (m, 0.16H), 7.20 (d, *J* = 9.3 Hz, 0.3H), 7.11 (s, 0.17H), 6.65 (d, *J* = 9.5 Hz, 0.3H), 6.58 (s, 0.33H), 3.87 – 3.47 (m, 24H), 3.21 (t, *J* = 5.1 Hz, 4H), 3.07 (dq, *J* = 14.1, 8.2, 6.9 Hz, 4H). ^31^P NMR (162 MHz, D_2_O) δ 0.85, -0.39, -0.42, -0.52, -0.57, -20.62, -20.79, -21.14, -21.24, -21.39, -21.56, -21.82, -22.40, -23.42.

^13^C NMR (101 MHz, D_2_O) δ 157.77, 131.57, 121.53, 114.12, 103.47, 71.14, 71.06, 69.57, 69.36, 68.67, 66.55, 54.91, 42.92, 41.52, 40.86, 40.77, 39.12, 38.26, 36.46, 23.67, 13.55.

Preparation of FITC-PCNSs. A solution of FITC-polyP (0.032 mmol, 225 mg) and Urea (0.75 mmol, 45.45 mg) in ddH_2_O (15 mL, pH 8) was prepared. Then, the system was ultrasonically dispersed for 30 min. A solution of CuSO_4_ (10.00 mmol, 1.596 g) in ddH_2_O (15 mL, pH 6.5) was stirred for 30 min at room temperature. After mixing the above solutions, transfer the mixture to a Teflon reactor and react at 60 °C for 12 h. The resulting blue product was then obtained by centrifugation and washed with ddH_2_O. The samples were lyophilized for 2 days.

Characterization of nanomaterials. The morphology of the FITC-PCNSs was determined by TEM (Hitachi HT7700) and SEM (Hitachi Regulus8100). The zeta potential of the nanomaterials in PBS was measured on a Nano-ZS instrument (Malvern Instruments Limited). The size of the nanomaterials in PBS was detected with a DLS instrument (Brookhaven BI-200SM). The vibration of the sample skeleton and infrared spectrum of the organic function were measured by a Fourier transform infrared spectrometer (Nicolet 7000-c, recording range: 400-4000 cm^-1^, resolution: 4 cm^-1^). The fluorescence emission spectra were measured on a fluorescence spectrophotometer (Shimadzu, RF-6000). The structure and composition were characterized using a Bruker D8 Advance powder diffractometer for XRD and a Thermo ESCALAB 250Xi spectroscope for XPS. High-resolution transmission electron microscopy (HRTEM) and energy-dispersive X-ray spectroscopy (EDXS) were performed on a JEOL JEM-F200 equipped with an X-ray energy-dispersive spectrometer. X-ray absorption fine structure (XAFS) characterizations at the Co K-edge (7709 eV) were collected with the beamline TLS07A1 station of the National Synchrotron Radiation Research Center (NSRRC) equipped with a sagittally focusing Si 111 monochromator. ^1^H NMR, ^13^C NMR, and ^31^P NMR spectra were obtained on a Bruker nuclear resonance (400 MHz) spectrometer. The concentration of Cu was measured by ICP-OES (Agilent 5110).

Evaluation of glycolytic capacity. Cells were seeded in 24-well plates at 2.5 × 10^4^ cells per well and cultured for 24 h. Control, FITC-PCNSs, CuCl_2_, and FITC-polyP treatment groups were established, with each treatment group further divided into oligomycin (+) and oligomycin (-) subgroups. The culture medium was removed, and the experimental groups were treated with FITC-PCNSs (25 μg mL^-1^), CuCl_2_ (100 μM), and FITC-polyP for 24 h (FITC-polyP was introduced into the cells via transfection, 0.057 μM, calculated). Following treatment, the medium was removed again, and the cells were gently washed three times with PBS. Subsequently, 200 µL of culture medium containing 1.25 µM oligomycin was added to the oligomycin (+) subgroup, while the oligomycin (-) subgroup received medium without oligomycin. The cells were incubated for an additional 3 h; 100 µL of culture medium was collected from each well, and the lactate in the medium was measured.

Assessment of lactate content. Lactate levels were determined by a Glycolysis/OXPHOS Assay Kit (DOJINDO LABORATORIES, G270). 20 µL of the sample was added to each well of a 96-well plate, followed by the addition of 80 µL of lactate working solution to each well. The mixture was incubated for 30 min, after which the absorbance was recorded at 450 nm.

Intracellular transfection and content assessment of FITC-polyP. We assessed the fluorescence intensity of various concentrations of FITC-polyP in the extracellular medium and subsequently plotted the relationship between fluorescence intensity and FITC-polyP concentration.

5 mg FITC-polyP was dissolved in 500 µL of medium. Subsequently, 8 µL of Lipo 2000 transfection reagent was added, and the mixture was gently mixed to achieve homogeneity. The cells were incubated with this medium for 24 h. The concentration of FITC-polyP internalized by the cells was assessed by measuring the fluorescence intensity within the cells.

Assessment of OCR. OCR was determined by the Extracellular OCR Plate Assay Kit (DOJINDO LABORATORIES, E-297). Cells were seeded in 96-well black plates at 3 × 10^4^ cells per well and cultured for 24 h. Blank1, Blank2, Blank3, control, and sample groups were established accordingly. Following overnight incubation, the wells designated for the sample treatment were exposed to FITC-polyP (0.057 μM, calculated), FITC-PCNSs (25 μg mL^-1^), and CuCl_2_ (100 μM) for 24 h. Subsequently, the medium was removed, and 100 µL of fresh medium was added to the wells of Blank1 and Blank3. In contrast, 100 µL of a working solution diluted from the oxygen probe was introduced into the wells of Blank2, control, and sample groups. After an additional incubation of 30 min, one drop of mineral oil was added to each well. The fluorescence intensity (E_X_ = 500 nm, E_m_ = 650 nm) was continuously read and detected at 10 min intervals for 200 min, and OCR was calculated.

Assessment of NAD^+^/NADH ratio. NAD^+^/NADH was determined by the NAD/NADH Assay Kit-WST (DOJINDO LABORATORIES, N509). Cells were seeded in 6-well black plates at 3 × 10^5^ cells per well and cultured for 24 h. The treatment was continued for 24 h with FITC-polyP (0.057 μM, calculated), FITC-PCNSs (25 μg mL^-1^), and CuCl_2_ (100 μM), respectively. Subsequently, the cells from each treatment group were collected, and further procedures were performed according to the manufacturer's instructions provided in the kit.

Cu^2+^ is released at different pH values. FITC-PCNSs (5 mg) were dispersed in 5 mL ddH_2_O at pH 5.5, 6.5, and 7.4, respectively. The solutions were dialyzed (MW=1000) in 30 mL of the respective ddH_2_O. Afterward, 1 mL of the above dialysis solution was taken out at regular intervals to quantify the Cu^2+^ concentration by ICP-OES.

GPC analysis. FITC-PCNSs (30 mg) were dispersed in 5 mL ddH_2_O at pH 5.5. The solutions were dialyzed (MW=1000) in 30 mL of respective ddH_2_O. After 24 h, the above retentate solution was collected and taken out to quantify the molecular weight by GPC.

^31^P NMR analysis. FITC-PCNSs (20 mg) were dispersed in 5 mL D_2_O at pH 5.5. The solutions were dialyzed (MW=1000) in 30 mL of respective D_2_O. Afterward, 500 μL of the retentate solution was taken out to measure ^31^P NMR.

Cell viability assay. Cells were seeded in 96-well plates at 1 × 10^4^ cells per well and cultured for 24 h. After the cells were treated with different concentrations of FITC-PCNSs for 24 h, 10 μL per well of CCK8 solution (KeyGEN, Nanjing, China) was added to the cells and incubated for an additional 4 h. Finally, OD_450_ was obtained with a microplate reader.

Western blot analysis. 4T1 cells were seeded in 6-well tissue culture plates at a density of 1.5 ×10^5^ cells mL^-1^ for 24 h. After treatment with FITC-polyP (0.057 μM, calculated), FITC-PCNSs (25 μg mL^-1^), and CuCl_2_ (100 μM) for 24 h, the cells were collected, and then the cell lysates were obtained with a Whole Cell Lysis Assay. The protein concentration was determined by the Super-Bradford Protein Assay Kit (CWBiotech, Inc., Beijing, China). Briefly, the extracts were first separated by 10% SDS‒PAGE and transferred to a polyvinylidene difluoride membrane (Bio-Rad, CA, USA). The membrane was blocked with 5% BSA in TBST at 25 °C for 1 h and then incubated with antibodies at 4 °C overnight. The expression of β-actin was used as the internal standard. Primary antibodies against the following proteins were used: β-actin (1:1000 for WB), PKM2 (1:1000 for WB), LDHA (1:1000 for WB), PFKM (1:1000 for WB), α-OGDH (1:1000 for WB), IDH2 (1:1000 for WB), PDHB (1:1000 for WB), IP3R (1:1000 for WB), MCU (1:1000 for WB), LIAS (1:1000 for WB), FDX1 (1:1000 for WB), DLAT (1:1000 for WB), ACO2 (1:1000 for WB), SDHB (1:1000 for WB). The appropriate secondary antibodies (1:1000 for WB) were purchased from CST. For the experiments, different target proteins were analyzed on separate membranes using aliquots from the same batch of protein lysates, with identical sample order and equal protein loading. β-Actin was analyzed in parallel on a separate membrane using the same set of lysates and served as a common loading reference for these parallel blots. For our earlier experiments, the transferred membrane was physically sectioned according to the expected molecular weights of the target proteins before antibody incubation. Therefore, a single antibody-exposed full-length membrane image does not exist for these experiments. We have provided all available original images of the corresponding membrane sections. For the additional Western blot data provided during the subsequent review, the membranes were left uncropped before antibody incubation, allowing us to provide full-length blot images for these experiments (Figure S59-73).

Quantitative RT-PCR. 4T1 cells were seeded in 6-well tissue culture plates at a density of 1.5 ×10^5^ cells mL^-1^ for 24 h. After treatment with FITC-PCNSs (25 μg mL^-1^), FITC-polyP (0.057 μM, calculated), and CuCl_2_ (100 μM) for 24 h, the total RNA of 4T1 cells with or without treatment was extracted using an E.Z.N.A.® Total RNA Kit I (R6834, OMEGA, USA) according to the manufacturer’s procedure. Total RNA concentration and purity were determined by the ratio of OD260/OD280 measured by a NanoDrop ND-2000 (Thermo Fisher Scientific, USA) instrument. Using the extracted total RNA as a template, cDNA synthesis was performed according to the FastKing cDNA First Strand Synthesis Kit (80328A, TIANGEN Biotech Co., Ltd, China). Primers for qPCR were designed with Primer Express. Subsequently, we added TB Green Premix Ex Taq II (KR116-02, Takara, China) (2×)5 μL, 2 μL of diluted cDNA, 0.4 μL of upstream and downstream primers (10 μM), 0.4 μL of ROX Reference Dye (50×), and deionized water to a total volume of 20 μL according to the instructions. Quantitative PCR was performed on triplicate samples in a 96-well format on the StepOnePlus™ Real-Time PCR Instrument (Applied Biosystems, Thermo Fisher Scientific, US) set to absolute quantification. The change in expression of a gene between the experimental and control conditions was computed using the ΔΔt method with actin as an internal reference gene^[52, 53]^.

The sequences are listed below:

LIAS-F: CCTGGGGTCCCGGATATTTG

LIAS-R: GAAGGTCTGGTCCATTATGCAA

DLAT-F: CTTTAGCCTCCAAAGCGAGAG

DLAT-R: AGATTGTAAATGTTCCACCCTGG

FDX1-F: CAAGGGGAAAATTGGCGACTC

FDX1-R: TTGGTCAGACAAACTTGGCAG

GAPDH-F: AGGTCGGTGTGAACGGATTTG

GAPDH-R: TGTAGACCATGTAGTTGAGGTCA

PKM2-F: GCCGCCTGGACATTGACTC

PKM2-R: CCATGAGAGAAATTCAGCCGAG

PFKM-F: TGTGGTCCGAGTTGGTATCTT

PFKM-R: GCACTTCCAATCACTGTGCC

LDHA-F: TGTCTCCAGCAAAGACTACTGT

LDHA-R: GACTGTACTTGACAATGTTGGGA

ICP-OES/MS Measurement Protocol. Transfer 50 μL of the prepared cell lysate into a 1.5 mL Eppendorf tube. Add 100 μL of concentrated nitric acid to the tube for digestion at 95°C for 1 h. After cooling, add 50 μL of H_2_O_2_ and continue incubation at 95°C for an additional 30 minutes. Following this step, adjust the total volume to 1 mL with ddH_2_O. Finally, measure the copper (Cu) content using inductively coupled plasma optical emission spectrometry/mass spectrometry (ICP-MS).

siRNA Transfection Analysis. To evaluate siRNA knockdown efficiency, 4T1 cells were seeded in T25 flasks and transfected when cell confluence reached approximately 30%. Following a 72-h transfection with siRNA-MCU, siRNA-FDX1, or siRNA-Nudt3, cells were washed, collected, and lysed using RIPA lysis buffer supplemented with a protease and phosphatase inhibitor cocktail. Protein concentrations were determined using the BCA protein assay kit. Equal amounts of protein were separated by SDS-PAGE and transferred onto nitrocellulose membranes. The membranes were subsequently probed with primary antibodies against β-actin (CST), MCU (CST), FDX1 (Abcam), and Nudt3 (CST). After incubation with species-matched horseradish peroxidase (HRP)-conjugated secondary antibodies, target protein bands were detected using an enhanced chemiluminescence (ECL) system. The sequences are listed below:

FDX1:

Sense: 5'- GGAUCACUCUUGAUAUUGATT -3'

Antisense: 5'- UCAAUAUCAAGAGUGAUCCTT -3'

Nudt3:

Sense: 5'- GCAUUGAGCUAGAUGAAGATT -3'

Antisense: 5'- UUCUUCAUCUAGCUCAAUGCTT -3'

MCU:

Sense: 5'- CUAUCAAGACAUUGAAGAATT -3'

Antisense: 5'- UUCUUCAAUGUCUUGAUAGTT -3'

*In Vitro* Cellular Uptake of FITC-PCNSs. We conducted a quantitative analysis of the internalization of FITC-PCNSs by cells over a defined time interval. 4T1 cells (1.2 × 10^6^ cells mL^−1^) were seeded in 6-well tissue culture plates and grown to ~90% confluence. Growth media in the plates were replaced with media containing FITC-PCNSs (25 μg mL^-1^). Cells were incubated with nanomaterials for 0, 3 h, 6 h, 9 h, 12 h, 18 h, and 24 h at 37 °C. Finally, the medium was removed from the wells, and the cells were washed three times with PBS to clear away nanomaterials outside of the cells. Next, 200 μL of 1% Triton X-100 in 0.1 M NaOH solution was added to lyse the cells. After treatment, Cu content in the lysates was determined using ICP-MS.

Intracellular Ca^2+^ imaging. 4T1 cells were seeded in 6-well tissue culture plates at a density of 1.5 ×10^5^ cells mL^-1^ for 24 h. Then, the cells were first treated with 25 μg mL^-1^ FITC-PCNSs for 24 h at 37 °C. Ca^2+^ concentration in the cells was determined using Rhod-2/AM (Thermo Fisher Scientific, USA) according to the method provided by the manufacturer. Ca^2+^ signals were analysed by an OLYMPUS SpinSR confocal laser scanning microscope equipped with multiple laser units.

Phase separation of the FITC-polyP/Ca^2+^ system. Commercially available heterogeneous polyP45 (also named “sodium phosphate glass”, Sigma-Aldrich) was dissolved in ddH_2_O to prepare 2× stock solutions at specified Pi concentrations (0.25, 0.5, 1, 10, 100, 200, and 400 μM Pi). Corresponding 2× Ca^2+^ stock solutions (concentrations ranging from 0.031 to 100 μM) were also prepared in this buffer. The Ca^2+^ concentrations were derived from the FITC-polyP to Ca^2+^ net charge ratio (R_-/+_), as detailed in Figure 4i. *In vitro* Ca^2+^ Phase separation was then performed by mixing equal volumes of FITC-polyP and Ca^2+^ stock solution, after which the mixture was transferred into a Petri dish and incubated at room temperature till the imaging process was finished within 4 h. Confocal images were taken on an OLYMPUS SpinSR confocal laser scanning microscope equipped with multiple laser units, a live cell chamber, and a built-in FRAP function. Images were captured using a UPLXAPO 100 × oil immersion objective and two channels of a cooled GaAsP PMT detector. Image analysis was performed with the manufacturer’s software, CellSens.

Fluorescence recovery after photobleaching (FRAP). For bleaching performed inside droplets, circular regions (1.5-2.0 mm diameter) in the centre of droplets (6.0-10.0 mm diameter) were selected as regions of interest (ROI). FITC was excited at 488 nm, and emission signals were collected in the range 500-520 nm. Ten images at attenuated laser intensity (20% intensity) were taken before photobleaching. Photobleaching was performed using a single pulse of an argon laser (405 nm) at 50% intensity for FITC (25 frames) through a round region of interest ROI. The laser was then switched back to attenuated intensity, and the recovery images were recorded for 100 seconds (200 frames). The time interval of each frame is 0.5 s. The recorded fluorescence intensities from the bleached ROI were then corrected for photofading and normalized using the FRAP function module of CellSens. Data fitting was performed with an exponential function f(t) = A [1 - exp(-t/t)] using GraphPad Prism.

Coacervate microdroplet fusion. The fusion tests between the coacervate microdroplets in aqueous solution were conducted by mixing the FITC-polyP and Ca^2+^ at a ratio of R_-/+_ = 16. The fusion processes of the polyP/Ca^2+^ coacervate microdroplets were monitored under bright-field and 488 nm fluorescence channels in an OLYMPUS SpinSR confocal laser scanning microscope, respectively.

Evaluation of the *in vivo* therapeutic efficacy of FITC-PCNSs. All animal experiments were performed following the National Institutes of Health animal care guidelines. Animal protocols were approved by the Institutional Animal Care and Use Committee (Nanjing University, IACUC-D2202146). To establish the tumor-bearing mouse model, female BALB/c mice purchased from the Model Animal Research Center of Nanjing University (Jiangsu, China) were subcutaneously injected into the left foreleg armpit of each mouse with 10^6^ 4T1 cells in 100 μL of PBS. Therapeutic interventions commenced when tumor volumes reached approximately 100 mm³, administered via tail vein injection (saline, FITC-polyP, FITC-PCNSs, or Cu-E) delivered on days 0, 3, 6, 9, 12, and 14. Systemic delivery protocols were concurrently implemented: FITC-polyP and FITC-PCNSs groups received intravenous injections at phosphorus-equivalent doses of 15 mg P/kg, while FITC-PCNSs and Cu-E cohorts were administered copper-equivalent doses of 10 mg Cu/kg via the same route. Initially, the mice were randomly divided into 4 groups (n = 8 per group). Over the 14 days treatment period, mice that died prior to the study endpoint due to tumor burden were excluded from the final end-point analyses. On day 14, tumors from all surviving mice were excised and weighed to assess the therapeutic efficacy. The tumor size and weight of all groups were measured once every two days. Tumor size was determined by a Vernier caliper at frequent intervals, and the tumor volume (*V*) was calculated by the formula:

$$V\left( {mm}^{3} \right)=\frac{L\times W^{2}}{2} (1)$$

where *L* is the length of the tumor and *W* is the width of the tumor. The mice were randomly divided into 4 groups (n = 8 per group). Moreover, the survival of tumor-bearing mice treated with different modalities was monitored. On day 14, the mice were euthanized, and the tumor tissue was collected and fixed with 10% neutral buffered formalin. Then, the tumors were embedded in paraffin, processed into 100 μm sections, and stained with hematoxylin and eosin (H&E). The images were obtained by digital microscopy.

Tumor metabolic reprogramming and cuproptosis sensitization assessment. At the end of treatment, tumor tissue was collected from each group for an immunofluorescence assay. Tumor tissues were fixed with 4% PFA and embedded in paraffin. The paraffin-embedded samples were sectioned into 5 μm thick histologic slides, which were blocked for endogenous activity with 2% H_2_O_2_ and pre-treated with Diva Decloaker buffer (Biocare Medical) for heat retrieval. The slides were washed with Tris-buffered saline with 0.025% Tween 20 detergent (TBST) and then blocked by incubation in Biocare Medical Background Sniper in 2% bovine serum albumin/PBS solution for 10 min. After removing the blocking buffer, anti-mouse monoclonal antibodies against PKM2 (1:50, Abcam), LDHA (1:50, Abcam), FDX1 (1:50, Abcam), DLAT (1:50, Abcam), and LIAS (1:50, Abcam) were applied to slides overnight at 4 °C in the dark. After re-staining with fluorescent secondary antibody and DAPI, the slices were imaged under a confocal laser scanning microscope (ZISSE). Lactate levels in tumour tissue fluid were measured using the Lactate Content Monitoring Kit (Solarbio).

*In vivo* toxicity of FITC-PCNSs. Blood samples were collected from mice across treatment groups for comprehensive hematological profiling and serum biochemical analysis. Hematological parameters were quantified using a Photoelectric MEK-8222K automated five-class blood cell analyzer. Serum biochemical markers were assessed with commercially available assay kits (Nanjing Jiancheng Bioengineering Institute, China). Major organs (liver, spleen, kidney, heart, and lung) were harvested and subjected to standardized histopathological processing: fixation in 10% neutral buffered formalin, paraffin embedding, sectioning, H&E staining, and subsequent digital microscopic evaluation.

Immune activation detection of FITC-PCNSs *in vivo*. The mouse model was established as described above, and tumor tissues were harvested on day 14 post-intervention. To maintain high cell viability for flow cytometry, fresh tumors from 3 randomly selected surviving mice per group (n = 3) were immediately harvested on day 14 and processed into single-cell suspensions. Single-cell suspensions underwent sequential immunophenotyping: initial staining with anti-CD3 FITC, anti-CD4 PE, and anti-CD8 APC antibodies for T-cell subset analysis via flow cytometry, followed by anti-CD3 FITC/anti-CD8a PE/anti-IFN-γ APC triple staining to quantify tumor-specific CD8^+^ effector T cells. Regulatory T cells (Tregs) were identified using anti-CD4 PE and anti-Foxp3 FITC dual labeling, supplemented with anti-CD25 APC for splenic Treg validation. Concurrently, serum cytokines including IL-6, IFN-γ and TNF-α were quantified using commercial ELISA kits.

Statistical Analysis. All quantitative data were processed without arbitrary exclusion or subjective transformation. Data normalization (e.g., percentage of control) was performed against untreated control groups. Exact sample sizes (n) for in vitro and in vivo cohorts are specified in the respective figure legends. All data are presented as the mean ± standard deviation (SD). Comparisons between two independent groups were performed using a two-tailed Student’s t-test. One-Way ANOVA was utilized for comparisons among three or more groups at a single time point, followed by Tukey’s post hoc test for multiple pairwise comparisons or Dunnett’s post hoc test for comparisons against a single control group. Tumor volumes were analyzed by Two-Way ANOVA with Tukey’s post hoc test. Cumulative survival curves were plotted using the Kaplan–Meier method and analyzed via the log-rank (Mantel–Cox) test. Statistical significance was set at *^*^p* < 0.05, *^**^p* < 0.01, *^***^p* < 0.001, and *^****^p*< 0.0001. “ns” indicates no significant difference. All analyses were performed using Prism version 10.0 (GraphPad, San Diego, USA).

Results

**Fig****ure S1.** Synthetic route to FITC-polyP.

**Figure S2.** ^1^H NMR spectrum of polyP-NH_2_.

**Figure S3.** ^31^P NMR spectrum of polyP-NH_2_.

**Figure S4.** ^13^C NMR spectrum of polyP-NH_2_.

**Figure S5.** ^1^H NMR spectrum of FITC-polyP.

**Figure S6.** ^31^P NMR spectrum of FITC-polyP.

**Figure S7.** ^13^C NMR spectrum of FITC-polyP.

**Figure S8.** SEM images and corresponding size distribution histograms of (a) FITC-polyP-Zn, (b) FITC-polyP-Mg, (c) FITC-polyP-Ga, (d) FITC-polyP-Mn, and (e) FITC-polyP-Ca. Scale bars represent 500 nm in Figures a–d and 200 nm in Figure e.

**Figure S9.** XPS analysis for (a) Cu *2p*, (b) O *1s*, (c) P *2p*, and (d) atomic ratio of FITC-PCNSs. Data points presented in Figure d were displayed as mean ± standard deviation (n = 3).

**Figure S10.** Morphological characterization of the PCNSs. (a) A typical SEM image of the PCNSs. Scale bar: 500 nm. (b) DLS analysis of the PCNSs. (c) Zeta potential of PCNSs. (d) Copper level per unit mass of nanostructures.

**Figure S11.** (a) The fluorescence emission spectrum of FITC-PCNSs (Ex = 445 nm). (b) Time-dependent Cu^2+^ release of FITC-PCNSs at different pH values. Data points presented in Figure b were displayed as mean ± standard deviation (n = 3).

**Figure S12.** ^31^P NMR spectra of polyP using 2 μL H_3_PO_4_ as the internal standard.

**Figure S13.** ^31^P NMR study monitoring the FITC-polyP released from FITC-PCNSs in 1 h using 2 μL H_3_PO_4_ as the internal standard.

**Figure S14.** ^31^P NMR study monitoring the FITC-polyP released from FITC-PCNSs in 3 h using 2 μL H_3_PO_4_ as the internal standard.

**Figure S15.** ^31^P NMR study monitoring the FITC-polyP released from FITC-PCNSs in 6 h using 2 μL H_3_PO_4_ as the internal standard.

**Figure S16.** ^31^P NMR study monitoring the FITC-polyP released from FITC-PCNSs in 12 h using 2 μL H_3_PO_4_ as the internal standard.

**Figure S17.** ^31^P NMR study monitoring the FITC-polyP released from FITC-PCNSs in 24 h using 2 μL H_3_PO_4_ as the internal standard.

**Figure S18.** ^31^P NMR study monitoring the FITC-polyP released from FITC-PCNSs in 36 h using 2 μL H_3_PO_4_ as the internal standard.

**Figure S19.** The ratio of the integral of FITC-polyP to the internal standard corresponding to Figures S12- 18. Data are displayed as mean ± standard deviation (n = 3).

**Figure S20.** Effect of FITC-polyP-Zn, FITC-polyP-Mg, FITC-polyP-Ga, FITC-polyP-Mn, and FITC-polyP-Ca on lactate secretion of (a) CT26, (b) A549, (c) U87, and (d) HeLa cells, respectively. All data are displayed as mean ± standard deviation (n = 3).

**Figure S21.** Toxicity of (a) FITC-polyP-Zn, (b) FITC-polyP-Mg, (c) FITC-polyP-Ga, (d) FITC-polyP-Mn, (e) FITC-polyP-Cu, and (f) FITC-polyP-Ca on 4T1, CT26, A549, U87, Hela cells, respectively. All data are displayed as mean ± standard deviation (n = 3).

**Figure S22.** ICP results of the 4T1 cells' uptake of FITC-PCNSs (25 μg mL^-1^). Data are displayed as mean ± standard deviation (n = 3).

**Figure S23.** Validation of the cellular uptake mechanism for FITC-PCNSs (25 μg mL^-1^). Pre-incubation of cells with the clathrin inhibitor Pitstop 2 (20 μM) for 1 h significantly inhibited the cellular uptake of FITC-PCNSs.

**Figure S24.** The subcellular distribution of FITC-PCNSs (25 μg mL^-1^) after treatment for 6 h. The extent of colocalization with mitochondria, ER, Golgi apparatus, and lysosomes was evaluated via Pearson’s correlation coefficient, with calculated R values of 0.34, 0.31, 0.39, and 0.75, respectively.

**Figure S25.** (a) The ratio of NADH/NADH + NAD^+^ after FITC-polyP (0.057 μM, calculated), CuCl_2_ (100 μM), and FITC-PCNSs (25 μg mL^-1^) treatment. (b) The ratio of NADH/NADH + NAD^+^ at different time points of FITC-PCNSs (25 μg mL^-1^) treatment. All data are displayed as mean ± standard deviation (n = 3). *^*^p* < 0.05, analyzed by One-Way ANOVA. “ns” indicates no significant difference.

**Figure S26.** CLSM image of FITC-polyP transfected cells (a) and corresponding fluorescence quantification (b). Figure b is displayed as mean ± standard deviation (n = 3). *^**^p* < 0.01, analyzed by Student’s t-test.

**Figure S27.** PCA plots from the whole proteomics of FITC-polyP (0.057 μM, calculated), FITC-PCNSs (25 μg mL^-1^), and CuCl_2_ (100 μM) treatment in 4T1 cells.

**Figure S28.** (a) Volcano plot of differentially expressed proteins in FITC-polyP treated 4T1 cells compared with the blank control. KEGG enrichment analysis of (b) downregulated and (c) upregulated proteins after FITC-polyP treatment.

**Figure S29.** CLSM images of Ca^2+^ in the cytoplasm after FITC-polyP (0.057 μM, calculated), FITC-PCNSs (25 μg mL^-1^), and CuCl_2_ (100 μM) treatments.

**Figure S30.** Relative mean FITC-polyP fluorescence intensity of CLSM images in Figure S29. All data are displayed as mean ± standard deviation (n = 3). *^**^p* < 0.01, *^***^p* < 0.001, analyzed by One-Way ANOVA. “ns” indicates no significant difference.

**Figure S31**. Intracellular localization of FITC-polyP/Ca^2+^ condensates. Colocalization analysis was performed for each organelle group. In the endoplasmic reticulum (ER) group, the PCC between FITC-polyP and Ca^2+^ (Rhod-2) was R = 0.86, and the PCC between FITC-polyP and the ER was R = 0.38. In the mitochondria group, the PCC between FITC-polyP and Ca^2+^ was R = 0.81, and the PCC between FITC-polyP and mitochondria was R = 0.29. In the lysosome group, the PCC between FITC-polyP and Ca^2+^ was R = 0.89, and the PCC between FITC-polyP and lysosomes was R = 0.31. The fluorescence intensity profiles further confirm the spatial overlap between FITC-polyP, Ca^2+^, and the respective organelles.

**Figure S32.** FITC-PCNSs exhibited no such fusion behavior after cellular internalization within a short time interval.

**Figure S33.** (a) CLSM images of different charge ratios R_-/+_ when the Pi concentration is 400 μM. (b) CLSM images of FITC-polyP co-localized with Ca^2+^ (Pi concentration is 400 μM, R_-/+_ =16).

**Figure S34.** Turbidity changes of LLPS occurring with FITC-polyP or polyP with Ca^2+^.

**Figure S35.** Influence of ionic strength on phase separation behavior.

**Figure S36.** (a) Standard curve of FITC-polyP concentration versus fluorescence intensity. (b) Intracellular release level of FITC-polyP in cells treated with FITC-PCNSs (25 μg mL^-1^) for 12 h. All data are displayed as mean ± standard deviation (n = 3). *^**^p <* 0.01*,* analyzed by Student’s t-test.

**Figure S37.** CLSM images of LLPS under a Pi concentration of 2.48 μM and R_-/+_ = 8.

**Figure S38.** Q-PCR analysed transcript levels of PFKM, PKM2, and LDHA. All data are displayed as mean ± standard deviation (n = 3).

**Figure S39.** CLSM images of phase separation in cells pretreated with 2-APB before FITC-PCNSs treatment.

**Figure S40.** Expression level of Nudt3 following siRNA-mediated Nudt3 knockdown. All data are displayed as mean ± standard deviation (n = 3). *^**^p <* 0.01*,* analyzed by Student’s t-test.

**Figure S41.** CLSM images of 4T1 cells sequentially treated with BAPTA (20 μM) for 6 h followed by FITC-PCNSs (25 μg mL^-1^) for 24 h.

**Figure S42.** CLSM images of 4T1 cells sequentially treated with FITC-PCNSs (25 μg mL^-1^) for 24 h followed by ALP (50 μg mL^-1^) for 6 h.

**Figure S43.** The pathway of ALP cellular internalization. Cy5 fluorophore was conjugated to ALP using a commercial Alkaline Phosphatase Labeling Kit - NH_2_. Significant intracellular fluorescence signals were observed following 6 h co-incubation of cells with ALP-Cy5 (50 μg mL^-1^). Conversely, when cells were pretreated with the clathrin-specific inhibitor Pitstop 2 (20 μM) for 1 h before ALP-Cy5 incubation, markedly reduced fluorescence signals were observed intracellularly.

**Figure S44**. Quantification of copper levels was performed by ICP-MS in cytoplasmic and mitochondrial fractions isolated from cells treated with FITC-PCNSs (25 μg mL^-1^) for the indicated durations. All data are displayed as mean ± standard deviation (n = 3).

**Table S2.** IC_50_ value (μg mL^-1^) of the FITC-PCNSs.

| Cell lines | IC_50_ (μg mL^-1^) |
| --- | --- |
|  | **FITC-PCNSs** |
| MRC-5 | 20.27 μg mL^-1^ |
| THP-1 | 16.88 μg mL^-1^ |
| HEK-293T | 21.56 μg mL^-1^ |
| 4T1 | 23.55 μg mL^-1^ |

**Figure S45.** Dose-response curves for cell viability and the corresponding IC50 values of FITC-PCNSs against (a) THP-1, (b) MRC-5, (c) HEK-293T, and (d) 4T1 cell lines. All data are presented as mean ± standard deviation (n = 3).

**Figure S46.** Q-PCR analysis of transcript levels for FDX1, DLAT (monomer), and LIAS. All data are displayed as mean ± standard deviation (n = 3).

**Figure S47. (a)** Expression level of FDX1 following siRNA-mediated FDX1 knockdown. (b) Cytotoxicity of FITC-PCNSs toward 4T1 cells following FDX1 knockdown. All data are displayed as mean ± standard deviation (n = 3). *^**^p <* 0.01*,* analyzed by Student’s t-test.

**Figure S48.** WB analysis of FITC-PCNSs (25 μg mL^-1^) treatment followed by 36 h of co-treatment with FITC-PCNSs and oligomycin (1.25 μM). All data are displayed as mean ± standard deviation (n = 3).

**Figure S49.** Immunofluorescence imaging analysis of FDX1, LIAS, and LDHA in tumor tissues from post-treatment Mice. Scale bar: 50 μm.

**Figure S50.** Quantitative fluorescence analysis corresponding to the fluorescence imaging in Figure S49: (a) FDX1; (b) LIAS; (c) DLAT. All data are displayed as mean ± standard deviation (n = 3). *^***^p* < 0.001, ^****^*p* < 0.0001, analyzed by One-Way ANOVA. “ns” indicates no significant difference.

**Figure S51.** Distribution of copper in different organs after administration. All data are displayed as mean ± standard deviation (n = 3).

**Figure S52.** Haematological analysis of mice after 14 days of treatment. All data are displayed as mean ± standard deviation (n = 3).

**Figure S53.** Biochemical analyses of mice after 14 days of treatment. All data are displayed as mean ± standard deviation (n = 3).

**Figure S54.** Pathological analysis of the spleen, liver, heart, lung, and kidney by H&E staining; scale bar: 100 μm.

**Figure S55.** Immunofluorescence imaging of (a) LDHA and (b) PKM2 in tumor tissues from treated mice, and corresponding quantitative fluorescence analysis of (c) LDHA and (d) PKM2. Scale bar: 100 μm. All data are displayed as mean ± standard deviation (n = 3). *^**^p* < 0.01, *^***^p* < 0.001, analyzed by Student’s t-test.

**Figure S56.** Reversal of FITC-PCNSs induced metabolic reprogramming and anti-tumor efficacy by the MCU inhibitor. (a) Tumor size determination after the indicated treatments. (b) Tumor weight of mice in the respective treatment groups (n = 5). (c) The body weights of the mice after the indicated treatments. (d) Lactate levels in the tumor tissues of mice across the different treatment groups. (e) Western blot images showing the protein expression levels of cuproptosis-related (LIAS, FDX1, and DLAT monomer) proteins in tumor tissues from the Saline, FITC-PCNSs, and FITC-PCNSs + MCU-i4 (5 mg/kg) groups (n=3). Quantitative analysis of the relative protein levels of (f) LIAS, (g) FDX1, and (h) DLAT based on the Western blot results. All data are displayed as mean ± standard deviation. *^*^p <* 0.05, *^**^p <* 0.01, *^***^p <* 0.001, and *^****^p <* 0.0001 by two-way ANOVA (a) or one-way ANOVA (b, d, f–h). “ns” indicates no significant difference.

**Figure S57.** CD8^+^/IFN-γ^+^ T cells in the TME by flow cytometry analysis. All data are displayed as mean ± standard deviation (n = 3). *^*^p* < 0.05, ^****^*p* < 0.0001, analyzed by One-Way ANOVA. “ns” indicates no significant difference.

**Figure S58.** CD4^+^/Foxp3^+^ T cells in the TME by flow cytometry analysis. All data are displayed as mean ± standard deviation (n = 3). *^*^p* < 0.05, *^**^p* < 0.01, analyzed by One-Way ANOVA. “ns” indicates no significant difference.

**Figure S59.** Original Western blot images corresponding to Figure 3b.

**Figure S60.** Original Western blot images corresponding to Figure 3k.

**Figure S61.** Original Western blot images corresponding to Figure 3m.

**Figure S62.** Original Western blot images corresponding to Figure 4a.

**Figure S63.** Original Western blot images corresponding to Figure 4c.

**Figure S64.** Original Western blot images corresponding to Figure 4e.

**Figure S65.** Original Western blot images corresponding to Figure 4g.

**Figure S66.** Original Western blot images corresponding to Figure 4i.

**Figure S67.** Original Western blot images corresponding to Figure 5g.

**Figure S68.** Original Western blot images corresponding to Figure 5i.

**Figure S69.** Original Western blot images corresponding to Figure 5k.

**Figure S70.** Original Western blot images corresponding to Figure S40.

**Figure S71.** Original Western blot images corresponding to Figure S47.

**Figure S72.** Original Western blot images corresponding to Figure S48.

**Figure S73.** Original Western blot images corresponding to Figure S56.

**Reference**

[51]	G. M. Fernandes-Cunha, C. J. McKinlay, J. R. Vargas, H. J. Jessen, R. M. Waymouth, and P. A. Wender, *ACS Central Science* **4**, no. 10 (2018): 1394–1402, https://doi.org/10.1021/acscentsci.8b00470.

[52] K. A. Kigerl, J. C. Gensel, D. P. Ankeny, Je. K. Alexander, D. J. Donnelly, and P. G. Popovich, *Journal of Neuroscience* **29**, no. 43 (2009): 13435-13444, https://doi.org/10.1523/JNEUROSCI.3257-09.2009.

[53] Q. Shi, A. T. Nguyen, Y. Angell, *et al*., *Gene Therapy* **17**, no. 9 (2010): 1085–1097, https://doi.org/10.1038/gt.2010.55.
